# Differentiation and Regulation of T_H_ Cells: A Balancing Act for Cancer Immunotherapy

**DOI:** 10.3389/fimmu.2021.669474

**Published:** 2021-05-03

**Authors:** Amrita Basu, Ganesan Ramamoorthi, Gabriella Albert, Corey Gallen, Amber Beyer, Colin Snyder, Gary Koski, Mary L. Disis, Brian J. Czerniecki, Krithika Kodumudi

**Affiliations:** ^1^ Clinical Science Division, Moffitt Cancer Center, Tampa, FL, United States; ^2^ Department of Biological Sciences, Kent State University, Kent, OH, United States; ^3^ UW Medicine Cancer Vaccine Institute, University of Washington, Seattle, WA, United States; ^4^ Department of Oncological Sciences, University of South Florida, Tampa, FL, United States; ^5^ Department of Breast Cancer Program, Moffitt Cancer Center, Tampa, FL, United States

**Keywords:** T helper, CD4, neoantigen, tumor associated antigen, immunotherapy

## Abstract

Current success of immunotherapy in cancer has drawn attention to the subsets of T_H_ cells in the tumor which are critical for activation of anti-tumor response either directly by themselves or by stimulating cytotoxic T cell activity. However, presence of immunosuppressive pro-tumorigenic T_H_ subsets in the tumor milieu further contributes to the complexity of regulation of T_H_ cell-mediated immune response. In this review, we present an overview of the multifaceted positive and negative effects of T_H_ cells, with an emphasis on regulation of different T_H_ cell subtypes by various immune cells, and how a delicate balance of contradictory signals can influence overall success of cancer immunotherapy. We focus on the regulatory network that encompasses dendritic cell-induced activation of CD4^+^ T_H_1 cells and subsequent priming of CD8^+^ cytotoxic T cells, along with intersecting anti-inflammatory and pro-tumorigenic T_H_2 cell activity. We further discuss how other tumor infiltrating immune cells such as immunostimulatory T_H_9 and T_fh_ cells, immunosuppressive T_reg_ cells, and the duality of T_H_17 function contribute to tip the balance of anti- vs pro-tumorigenic T_H_ responses in the tumor. We highlight the developing knowledge of CD4^+^ T_H_1 immune response against neoantigens/oncodrivers, impact of current immunotherapy strategies on CD4^+^ T_H_1 immunity, and how opposing action of T_H_ cell subtypes can be explored further to amplify immunotherapy success in patients. Understanding the nuances of CD4^+^ T_H_ cells regulation and the molecular framework undergirding the balancing act between anti- vs pro-tumorigenic T_H_ subtypes is critical for rational designing of immunotherapies that can bypass therapeutic escape to maximize the potential of immunotherapy.

## CD4^+^ T Cells Classification

As immunotherapy emerges as an effective therapeutic strategy in cancer, T helper (T_H_) cells have received widespread interest owing to their integral role in anti-tumor immune responses as has been demonstrated by diverse pre-clinical and clinical models (1, 2). While CD8^+^ cytotoxic T lymphocyte (CTL) function has been explored extensively in recent years in the context of immunotherapy (3), research shows the crucial role of CD4^+^ T_H_ cells and its interaction with dendritic cells (DC) to transmit necessary molecular help that stimulates CTL function (4). T_H_1 and T_H_2 subclasses of helper T cells engage in molecular crosstalk with multiple immune signaling pathways and have been investigated for their immunotherapeutic relevance in cancer. Considering the multidimensional role of CD4^+^ T_H_ cells, it is of utmost importance to understand the biology of these cells and how they contribute to tumor immune responses. Non-naïve CD4^+^ T cells are categorized as either effector or memory T cells. CD4^+^ memory T (T_M_) cells constitute a subpopulation of CD4^+^ T cells crucial in the immune system response against infections and non-infectious antigen exposure. Detailed mechanisms of differentiation and function of each T_M_ cell subtype is not discussed in this review, since it has been extensively reviewed elsewhere (5, 6). T_M_ cells are broadly subclassified into T_RM_ (tissue- resident memory cells) cells which are thought to reside specifically in the area of previously infected tissue, while T_CM_ (T central memory) and T_EM_ (T effector memory) cells are found circulating in the blood (both subtypes), lymphoid organs (T_CM_ cells) and peripheral organs (T_EM_ cells) (7), and overall, has been shown to be critical for eliciting anti-tumor immune response (8).

### CD4^+^ T Cells in Cancer

While distinct surface marker and functional profiles set T_M_ cell subtypes apart, T_RM_ cells have been crucial in anti-tumor immunity since a T_RM_ cell signature in the tumor has been associated with favorable prognosis in terms of disease-free survival and overall survival in breast cancer, ovarian cancer, cervical cancer, melanoma, lung cancer, head and neck squamous cell carcinoma, gastric cancer, bladder cancer and pancreatic cancer (5). Along with the expression of CD103 (integrin-αE) and CD69 surface markers, expression of immune checkpoint regulator genes such as PD-1, CTLA-4, TIM-3 and LAG-1 on T_RM_ cells obtained from solid tumors, clonal expansion of PD-1^+^TIM-3^+^ T_RM_ cells with high expression of proliferation and cytotoxicity markers, and enrichment of this specific cell type in lung cancer patients responding to PD-1 antibody therapy (9) suggest T_RM_ cells are a promising target for checkpoint inhibitor antibodies to offer therapeutic benefit in a myriad of solid tumor types.

In >300 patients with early stage triple negative breast cancer (TNBC), Savas et al. identified a gene signature of T_RM_ cells (high expression of the integrin αEβ7 αE chain (CD103) and significantly lower expression of SELL, KLRG1, KLF2, S1PR1 and S1PR5 genes) by single cell sequencing that shows significant positive association with reduced risk of recurrence and overall survival (10). In TNBC patients receiving combination therapy of chemotherapy with immunotherapy, specifically pembrolizumab, and/or targeted therapy, T_RM_ cell gene signature was associated with higher pathological complete response rate (pCR) in the I-SPY 2 neoadjuvant trials with 989 patients (11) and in the KEYNOTE-086 trial, in 200 patients with advanced-stage TNBC receiving pembrolizumab monotherapy (12–14). Compared to their CD8^+^ counterparts, CD4^+^ T_M_ cells appear to be persistent and regulated separately, independent of previous antigen exposure or homeostatic mechanisms (15). In the context of cancer therapy, long-lasting response to tumor antigen is critical, hence the importance of developing immunotherapies that stimulate these responses *via* CD4^+^ T_M_ cells.

## T_H_ Cells: Functional Classification

**Figure 1:**
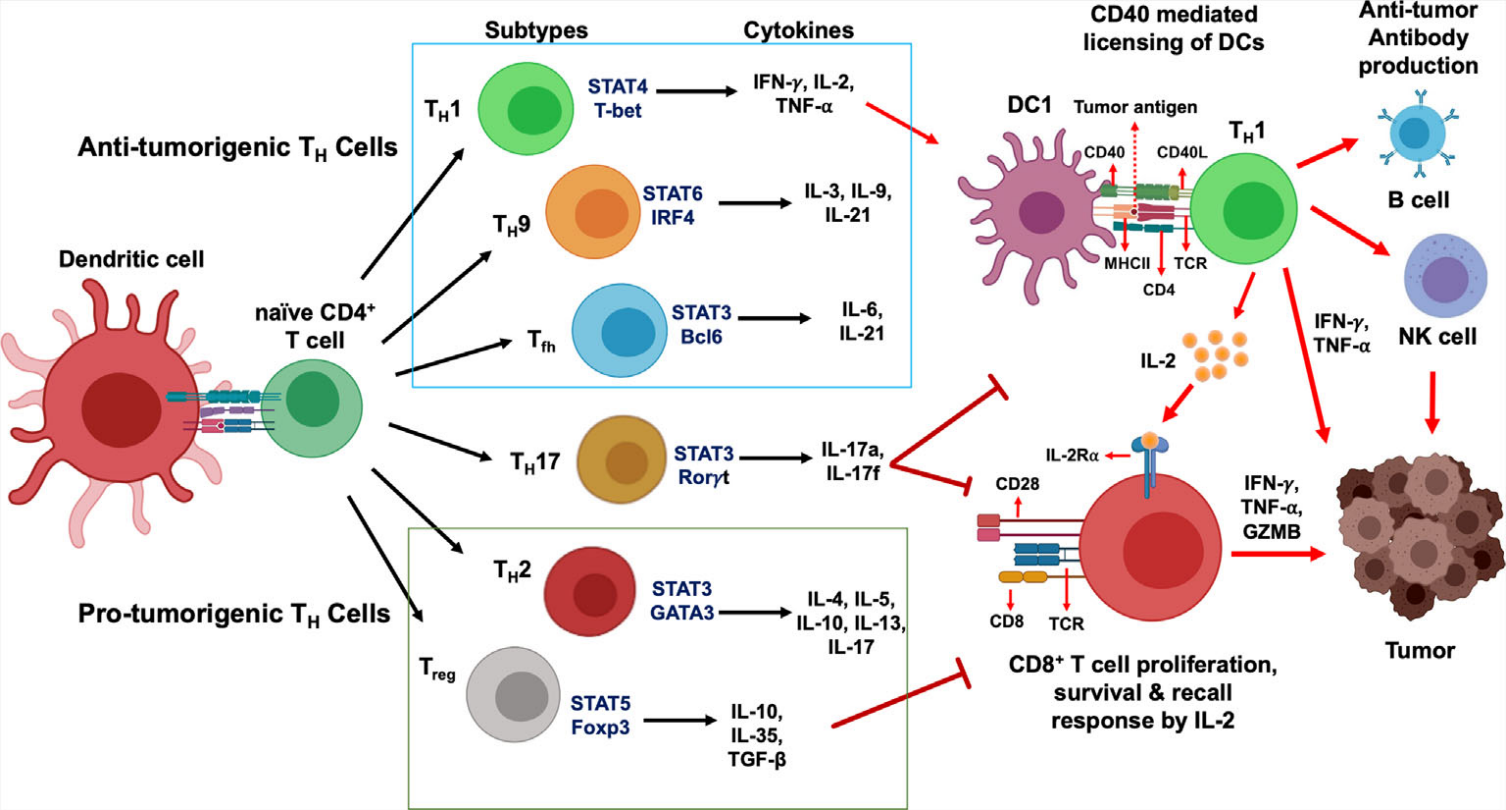
CD4+ T cells development and their functional subsets in immunity. T cell receptor (TCR) signaling activation, co-stimulation and presence of specific cytokines milieu have been shown to stimulate naïve CD4^+^ T cells polarization and their differentiation into T_H_1, T_H_2, T_H_9, T_H_17, T_fh_ and T_reg_ cell subtypes. While T_H_1, T_H_9 and T_fh_ cells (*green box*) stimulate anti-tumor immune response, T_H_2 and T_reg_ cells (*red box)* induce immunosuppressive protumorigenic response and a dual role of T_H_17 cells contribute to the functional complexity of this network. Primary STAT signaling pathways and major transcription factors regulating CD4^+^ T cell subtype polarization and key effector cytokines secreted from each Th cell subtypes are depicted. Dendritic cells present tumor antigenic peptides to T_H_1 cells *via* MHC class II molecule, leading to T_H_1 immune response activation. IFN-γ and TNF-α secreted from activated T_H_1 cells directly act on tumor cells and induce apoptosis, senescence, and proliferation arrest. In addition, T_H_1 cells can upregulate NK cells and B cells activation to further strengthen anti-tumor immune responses. Activated T_H_1 cells secrete IL-2 which mediates direct activation of CD8^+^ T cells expressing IL-2Rα and their proliferation and survival. In contrast, T_reg_ cells and T_H_17 cells are known to exhibit an immunosuppressive microenvironment that promotes tumor progression.

Activated CD4^+^ T cells differentiate into several functional classes based on the cytokine milieu, antigen presentation, and expression of costimulatory molecules. Combinations of environmental stimuli and autocrine cytokine production lead to the induction of several signaling pathways to regulate the expression of lineage-specific transcription factors. CD4^+^ T_H_ cells are polarized to one of several effector types defined by cytokine profiles and immune functions: T_H_1, T_H_2, T_H_17, T_H_9, T_reg_, and T_fh_ (16–20). Here we discuss the differentiation and secretion profile of each T_H_ cell subtypes (**Figure 1**), before delving deep into the molecular mechanism of signaling crosstalk between DC, CD4^+^ and CD8^+^ T cells and its therapeutic implication.

### T_H_1 Immune Response

T helper type 1 (T_H_1) and type 2 (T_H_2) are the two predominant classes of CD4^+^ T_H_ cells and were the first to be characterized by the production of interferon gamma (IFN-γ) and interleukin-4 (IL-4) cytokines, respectively (18). Specifically, generation of a T_H_1 effector subset is dependent on IL-12 and IFN-γ cytokines. IL-12 recruits natural killer (NK) cells to produce IFN-γ and together leads to activation of the signal transducer and activator of transcription-1 (STAT1) and STAT4 signaling pathways to induce the expression of the major transcription factor T-box expressed in T cells (T-bet), which drives T_H_1 differentiation by suppressing T_H_2/T_H_17 differentiation (17–20). Positive feedback regulation by IFN-γ secreted from these CD4^+^ T_H_1 cells support further T_H_1 differentiation (18, 19). The major cytokines and chemokines secreted from T_H_1 immune cells are the primary effector molecules downstream of immune cell signaling and will be discussed in detail later in this review.

### T_H_2 Immune Response

Polarization to the T_H_2 effector lineage is dependent on production of IL-4, stimulating STAT6 signaling to upregulate the GATA3 transcription factor (18, 20). Similar to T_H_1 differentiation, a positive feedback loop is supported by autocrine IL-4 secreted from T_H_2 cells, while combined IL-6 production by antigen presenting cells and GATA3 expression suppresses T_H_1 differentiation (17–20). The balance between IFN-γ and IL-4 feedback loops is critical to the balancing act between T_H_1 and T_H_2 CD4^+^ T cell immune responses.

T_H_2 differentiation has been shown to be dependent on IL-4 signaling *via* STAT6 signaling and transcriptional upregulation (21, 22). Once believed to solely derive from T_H_2 cells, IL-4 has since been known to be secreted by B cells, natural killer T cells (NKT), naïve CD4^+^ T cells and mast cells that can induce T_H_2 differentiation (23). Regulation of T_H_2 differentiation and cytokine profile has been comprehensively discussed previously (22, 24, 25). Binding of IL-4 to IL-4 receptors on immune cells leads to STAT6 phosphorylation, nuclear translocation, and expression of GATA3 transcription factor, resulting in T_H_2 cytokine secretion and eventual tumor growth and metastasis (26, 27). In studies ranging from lymphoma, melanoma, colorectal, breast, and lung cancer, STAT6 is overexpressed within the tumor microenvironment (TME) as an immunosuppressive signal to promote the function of M2 macrophages to assist in tumor growth and inflammation (28). To prevent dominance over each other, IL-12 expression from T_H_1 cells inhibits the differentiation of T_H_2 cells, while IL-4 inhibits T_H_1 differentiation (29).

Following differentiation, T_H_2 cells secrete IL-4, IL-5, IL-10, IL-13, and IL-17, not all of which are beneficial in cancer and have been shown to contribute to the tumor promoting role of this subtype. While IL-4, IL-5 and IL-13 have been documented to contribute to cancer growth and metastasis (21, 30, 31), a dual pro- and anti-tumorigenic role of IL-10 has been reported in recent literature, as reviewed elsewhere (32, 33). IL-10 elicits an anti-inflammatory immune response, downregulates T_H_1 cytokine function and MHC class II antigen presentation (29). Simultaneously, binding of IL-10 with its cognate receptor activates STAT3 signaling and transcription of anti-apoptosis and cell cycle progression genes that further strengthen the protumorigenic effect (34).

### T_H_9 Immune Response

Expanding our view of CD4^+^ T_H_1 and T_H_2 cells, there are some less explored T_H_ cell subsets which have unique potential in adaptive anti-tumor immunity. T_H_9 CD4^+^ T cells were once believed to be included within the T_H_2 subset, before being recognized as an individual population (35). Differentiation from naïve T cells to T_H_9 cells is facilitated by TGF-β and IL-4, mostly secreted from T_H_2 cells and these T_H_9 cells can stimulate uptake and presentation of antigens by DC for CD8^+^ T cell activation by secretion of IL-9 and signaling *via* CCL20-CCR6 axis (36, 37). While the functional profile of T_H_9 cells appears like that of T_H_1 cells, T_H_9 cells were found to be less exhausted in the TME of lung carcinoma patients (38). This could offer a possible improvement to immunotherapy treatments if T_H_9 cell proliferation can be increased, driven by the secretion of CCL20-CCR6 and IL-3. In tumor models, activation of this CCL20-CCR6 axis by T_H_9 significantly drives DC generation and the proliferation of CD8^+^ T cells to attack cancer cells in the TME (35). IL-3 is also involved in the prevention of DC apoptosis, allowing prolonged CTL activation within draining lymph nodes (36). IL-21 secretion by T_H_9 has also been shown to increase during anti-tumor response, which stimulates IFN-γ production by CD4^+^ T cells and is also involved in NK cell activation (36). An adoptive cell therapy study comparing the effects of T_H_1, T_H_17, and T_H_9 cell transfer determined that T_H_9 can induce a powerful enough immune response to fully regress B16 melanoma tumors in C57BL/6 mice. T_H_9 cells outcompeted both T_H_1 and T_H_17 responses, both of which were able to temporarily regress the tumor, but eventually succumbed to tumor growth relapse (38).

T_H_9 effector differentiation results from STAT6 signaling to express the IRF-4 transcription factor through TGF-β and IL-4 cytokine production (17, 20). Significance of Notch1 developmental signaling to induce T_H_9 differentiation has been investigated in recent years. Notch1a activates the transcription factor SMAD3, which binds to the IL-9 cytokine promotor, and increases T_H_9 proliferation (35). The primary concern regarding T_H_9 function in the TME is the inconsistency within various tumor types. While the increase of the CCL20-CCR6 interaction is beneficial in antigen uptake by DC, the expression of CCL20 can also promote tumor cell migration as seen in a study involving lung carcinoma. IL-9 secretion can suppress immune cell response as well. However, a study found that the neutralization of secreted IL-9 limits the effect to only migration of the immune cells without affecting other immune functions (39).

As previously stated, T_H_2 and T_H_9 cells share many transcription factors involved in the differentiation of these subsets. While T_H_2 and T_H_9 both share the STAT6 signaling pathway, there are some other transcriptional differences that set them apart. For instance, PU.1, part of the EST family of transcription factors, is more highly expressed in T_H_9 cells compared with T_H_2 cells and is linked to targeted IL-9 secretion from T_H_9 cells, while constraining T_H_2 differentiation. On the same note, the IL-4R-STAT6-GATA3 axis in T_H_9 cells is functionally different than in T_H_2 cells. In T_H_9 cells, its role is to act on FoxP3 expression induced by TGF-β, while the same axis drives IL-4 expression from T_H_2 subsets (36). Therefore, despite shared transcription factors between the subsets of T_H_ cells, each one has a distinct role to play in one subset that is separate from the other, leading to polarization and functional differences.

### T_H_17 Immune Response

Following T_H_1 and T_H_2 effector classifications was the recognition of T_H_17 and regulatory T cells (T_reg_), which differentiate through similar cytokine production profiles. T_H_17 lineage is characterized by the production of IL-17A-F, IL-21, IL-22, IL-10, IL23, and CCL20. Polarization proceeds through three stages with TGF-β and IL-6 driving T_H_17 differentiation *via* STAT3 activation and expression of major transcription factor RORγt (18–20). Autocrine amplification by IL-21 production and secretion of IL-23 from antigen presenting cells (APC) stabilizes the T_H_17 lineage (19, 20). IRF-4 is also important for T_H_17 subtype induction, amplification and stabilization by IL-21 and subsequent IL-17 production (16). While TGF-β is important to T_H_17 differentiation, high concentrations of TGF-β can result in the activation of STAT5 signaling and upregulation of the FOXP3 transcription factor to drive T_reg_ differentiation (18, 20). T_H_17 immune cells display plasticity during immune response and induce immune regulatory functions (40), contributing to impaired immune functions by targeting granzyme B production, a dominant marker for cytolytic CD4^+^ activity (1, 41).

In the context of tumor immune response, T_H_17 cells can not only use these similarities to T_H_1 as an effector memory cell, but its stem-like properties can allow them to elicit immune response for a longer duration than T_H_1 cells, positing the question of further research into their future use in cellular immunotherapy (42, 43). During tumor development, T_H_17 promoting chemokines and cytokines are expressed within the TME, such as CCL4, CCL17, CCL22, IL-1β, IL-6, IL-23, and TGF-β (44). While T_H_17 exhibits anti-tumor immune responses, the increase of these promotors is driven by tumor-associated macrophages (TAM) within the tumor to assist with tumor growth. Evidence of this can be found in melanoma, breast, ovarian, hepatocellular, pancreatic, and renal cancers and can be attributed to the role of cytokine IL-17 in angiogenesis by increasing VEGF and IL-6 production and myeloid-derived suppressor cells (MDSC) production resulting in immunosuppression within the tumor (45).

### T Follicular Helper Cells

T follicular helper cells (T_fh_) are considered the fifth major lineage of CD4^+^ T_H_ cells and are involved in the generation of high-affinity antibody responses by supporting B cell proliferation and helping to facilitate immunoglobulin class switching (19). The production of IL-6 and IL-21 induces the expression of the Bcl-6 transcription factor through STAT3 signaling and leads to the polarization to a T_fh_ effector class (19, 20). In systemically untreated breast cancer patients, CD4^+^ T cells were found to be the principal component of the tumor infiltrating lymphocytes (TIL) and along with T_H_1, T_H_2 and T_H_17 subtypes, were also enriched for T_fh_ populations (46). Purified CD4^+^ T cells from a cohort of non-small-cell lung carcinoma (NSCLC) patients showed a T_fh_ signature associated with heightened CTL proliferation and adoptive transfer of T_fh_ cells in a murine tumor model augmented CTL function and inhibited tumor growth *in vivo* (47). Expression of ICOS and PD-1 as markers of activated T_fh_ cells in breast cancer has been reported while RNA analysis showed enhanced expression of IL-21, IFN-γ, and CXCL13 on sorted T_fh_ TIL, and only ICOS^+^PD-1^+^T_fh_ TIL from HER2^+^ and triple negative breast cancer were capable of inducing *in vitro* IgG secretion by B cells (46). Details of T_fh_ cells differentiation, signaling and functional profile has been reviewed comprehensively elsewhere (48, 49).

### Regulatory T Cells

CD4^+^ regulatory T (T_reg_) cells exhibit critical roles in maintaining self-tolerance and preventing various autoimmune diseases. In contrast, T_reg_ cells also play a detrimental role in promoting cancer progression *via* regulating immune surveillance and suppressing anti-tumor immune response (50). Elevated levels of T_reg_ cells is associated with disease progression and poor survival in patients with various types of cancer (51, 52) as it is postulated that the reduced efficacy of various targeted therapies and immunotherapies is hindered by the activation of T_reg_ cells. T_reg_ cells has the ability to limit the function of antigen presenting cells by CTLA-4 dependent downregulation of CD80 and CD86 expressions, thereby evading tumor antigen presentation and tumor-specific T cell activation (53). In addition, the interlink between the expression of PD-1 on T_reg_ cells was observed as a negative regulator, highlighting that PD-1 blockade therapy may not only recover dysfunctional CD8^+^ T cells, but also enhances the suppressive function of T cells in cancer (54, 55). T_reg_ cells may also regulate the anti-tumor effects of T cells *via* the secretion of important immune suppressive cytokines such as IL-10, IL-35 and TGF-β (56). TGF-β secretion from T_reg_ cells can regulate CTL function and reduce anti-tumor immunity, since an almost complete suppression of CD8-mediated cytolytic activity was found to be essentially dependent on TGF-β signaling, and CD8^+^ cells with a dominant negative TGF-β receptor were resistant to this suppression (57). Previous studies have shown that T_reg_ cells can prevent CD4^+^ and CD8^+^ T cells proliferation and function by decreasing the availability of IL-2 (58). STAT5 signaling activation in T_reg_ cells utilizing IL-2/IL-2 receptor signaling is necessary to acquire immunosuppressive function and control CD8^+^ T cell expansion (59). A recent study investigated the antigen specificity for T_reg_ cells in metastatic melanoma, gastrointestinal cancer and ovarian cancer and found that intratumoral T_reg_ cells reacted specifically to tumor antigen, resulting in activation and clonal expansion of T_reg_ cells (60).

## Role of DC in CD4^+^ T_H_ Cell Differentiation and Function

DC can be characterized as conventional (cDC) and plasmacytoid DC (pDC) where classical DC include all DC except pDC even though they are derived from the same origin (2, 23, 61). Our lab has previously shown calcium mobilization induces mature, activated DC phenotype acquisition (i.e. CD83^+^and costimulatory molecule expression) and antigen sensitization in T cells through an apparently calmodulin-dependent mechanism in normal and transformed myeloid-derived cells (62). Our research group has also reported that in human PBMC-derived myeloid DC, presence of a calcium ionophore during DC maturation step antagonized IL-12 secretion in a calcineurin phosphatase-independent manner and showed preferential ability for T_H_2 polarization (63). The inherent plasticity of DC further segregates these functional classes based on expression of surface receptors, secreted stimuli, and migratory capabilities. Plasmacytoid DC express surface markers including CD123, CD202, CD303 and CD304, which are absent from the surface of cDC, and function to monitor viral infections with capacity to secrete IFN-α and IFN-β (64, 65). Specifically, cDC are known for antigen presentation and classified as cDC1 and cDC2 based on functional activity, activation of adaptive T-cell response, and expression of MHC-I and MHC-II, respectively (2, 23, 61, 66). cDC1 express transcription factors IRF8, Btaf3, and Id2 in both human and mouse and exhibit CD141, CLEC9a, CADM1, BTLA, CD26, and XCR1 surface expression; while cDC2 polarization is driven by IRF4 and ZEB2 transcription factors and primarily express CD1c, CD11b, CD11c, CD2, FCεR1, SIRPA, and CLECL10A (64, 67, 68). As in humans, mice also demonstrate phenotypic distinction between cDC1 and cDC2 by CD8α+ (lymphoid) and CD103+ (migratory) expression on cDC1, and CD4+ (lymphoid) and CD11b+ (migratory) expression on cDC2 (65, 68, 69).

### Role of DC in T_H_1 and T_H_2 Differentiation

Functionally, cDC1 are involved in antigen cross-presentation to stimulate CD8^+^ T cell cytotoxicity, and additionally play a role in CD4^+^ T_H_1 differentiation and recruitment of NK cells through IL-12 production (23, 61). Expression of Notch ligand delta on DC upon LPS exposure has also been shown to stimulate T_H_1 polarization, whereas exposure of DC to cAMP up regulators such as prostaglandin-E2 can direct CD4^+^ T cells towards a T_H_2 phenotype in an IL-4 independent manner *via* expression of notch ligand Jagged. Similarly, CD70 expressed on mouse DEC-205+ DC can act as a T_H_1 phenotype inducer, independent of IL-12 (70). Conventional DC activating CD4^+^ T cells is a controversial topic where recent studies have implicated cDC1 as being capable of activating CD4^+^ T cell responses in cancer (2, 61, 64, 71, 72); however, previously it has been understood that cDC1 secrete lower levels of IL-12 in comparison to cDC2, and cDC2 are recognized as the predominant activators of CD4^+^ T cell anti-tumor immunity (23, 64, 67, 72). This is in line with observations demonstrating that cDC2 are better equipped for CD4^+^ T cell differentiation due to preferential expression of MHC-II (64, 72, 73). Additionally, past research has demonstrated the preferential activation of CD8^+^ cytotoxic T cells by cDC1 through studies with Batf3-deficient mice unable to reject highly immunogenic tumor cell lines (74), while cDC2 have the capacity to stimulate CD4^+^ T cell differentiation and polarization into T_H_1, T_H_2, and T_H_17 effector populations *via* production of an array of cytokines such as IL-23, IL-1, TNF-α, IL-6, and IL-10, cytokines (64, 67).

### Role of DC in T_H_9 Differentiation

Dectin-1 is a β-glucan receptor present on DC, macrophages and neutrophils and dectin-1-activated DC have been shown to secrete IL-6, IL-12p40 and TNF-α, leading to T_H_1 and T_H_17 polarization (75). However, Zhao et al. have described dectin-1-activated DC promote a potent T_H_9 polarization *in vitro* by overexpression of TNFSF15 and OX40L *via* Syk, Raf1 and NF-κB signaling pathways. While they observed a significant increase in IL-9 and T_H_9-associated transcription factor IRF4, no changes were observed for other T_H_ subtype-associated cytokines and transcription factors *in vivo.* Anti-tumor effects of Dectin-1-activated DC in melanoma and myeloma preclinical model relied heavily on this T_H_9/IL-9 response while microarray analysis identified more than 40 cytokines, chemokines and costimulatory molecules such as TNF-a, TNFSF15, OX40L, TNFSF8 and low IL-12 (76, 77).

### Role of DC in T_H_17 Differentiation

Ability to produce cytokines IL-6, TGF-β, IL-1b and IL-23 support a critical role of DC in polarizing T_H_17 phenotype, as observed *ex vivo* in human DC isolated from inflammatory fluids, equivalent to monocyte-derived DC in mice. Likewise, monocyte-derived DC cultured *in vitro* with lymphoid tissue-resident bacteria secrete T_H_17-polarizing cytokines (78). In an experimental allergic encephalitis mouse model, CD11b^+^ myeloid DC in the central nervous system produce IL-23, TGF-β and IL-6, thereby inducing T_H_17 cells. Similarly, stimulation of human monocyte-derived DC with intact *E. coli* and ATP stimulates IL-23 secretion that further activates IL-17 producing T_H_17 cells (70). Significance of DC differentiation and antigen exposure on T_H_ cell polarization has been further highlighted in a study by Khayrullina et al. as DC differentiated in presence of prostaglandin E2 promotes an IL-17-producing T_H_17 phenotype by inducing a modified IL-12/IL-23 balance and inhibition of T_H_1/T_H_2 polarization, both *in vitro* and *in vivo*.

### Role of DC in T_fh_ Differentiation

Co-operation between DC and B cells induces and ensures differentiation into T_fh_ phenotype and lymph node-resident cDC2s in van Gogh mouse model has been shown to be sufficient for such T_fh_ priming. The unique localization of cDC2 in the interfollicular zone at the T cell-B cell border makes them ideally positioned to be the dominant T_fh_-priming DC subset in both human and mouse, which is also consistent with preferential antigen presentation on MHC-II by cDC2s and stronger antigenic stimulation favoring T_fh_ cell differentiation. Mice deficient in cDC2 (Cd11cCre Irf4^−/−^and Cd47^−/−^), but not cDC1 (Batf3^−/−^), demonstrate impaired T_fh_ responses to sheep RBCs and loss of DC in the T cell-B cell border leads to loss of T_fh_ polarization as well. However, cDC2 is not the sole determinant of the T_fh_ phenotype as a specific subset of cDC2 dependent on transcription factor krueppel-like factor 4 (KLF4) and expressing CD301b can induce only T_H_2 but not T_fh_ polarization, highlighting the diversity of T cell fate determinants, that also includes recruitment of various cytokines such as IL-6, IL-12 and IL-21 not secreted by cDC2s (61, 79).

The role of DC to selectively develop adaptive regulatory T cells has been highlighted as an inducer of peripheral tolerance. Simultaneously, negative regulation by IL-10 resulting in downregulation of MHC-II expression, IL-12 secretion and maturation of DC leads to an indirect preference for immune tolerance, and can induce regulatory DC that promote an IL-10-producing T_reg_ phenotype (80).

Studies have noted the contribution of antigenic density in determining CD4^+^ T cell fate where higher antigen doses are associated with T_H_1 differentiation in contrast to lower antigen doses leading to T_H_2 differentiation (23, 61). Overall, antigenic stimulation combined with simultaneous interactions between costimulatory molecules and cytokine stimuli produced by DC induce downstream signaling pathways that lead to T cell effector differentiation as discussed above. As tumor cells more readily express MHC-I molecules, DC play a pivotal role in the activation and priming of CD4^+^ T cells to initiate the CD4^+^ anti-tumor response.

## Molecular ‘HELP’ by CD4^+^ T_H_ Cells are Necessary for Cytotoxic Function of CD8^+^ T Cells

Cytotoxic and memory CD8^+^ T cell response as a principal component of immunity requires priming and expansion, both of which demand active help by CD4^+^ T cells. Even though the supporting role of CD4^+^ T_H_ cells to promote effector and memory function of CD8^+^ T cells have been well-established by late 1990s, recent research have generated crucial supporting evidence of the necessity of CD4^+^ T_H_ cells for anti-tumor CD8^+^ T cell function (81, 82). Neoantigen-specific vaccination has often showed largely CD4^+^ T cell response, and not CD8^+^ T cell response, in multiple pre-clinical models and clinical trials. In MMTV-PyMT spontaneous mammary carcinoma model, a unique T_H_1 CD4^+^ subset was identified in non-tumor peripheral tissues that rendered protective benefit when transferred into treatment-naïve tumor hosts challenged with 4T1 tumors (83). In an aggressive B16F10 murine melanoma model, IL-21 secretion stimulated by CD4^+^ T_H_ cells drives CD8^+^ T cell differentiation towards CX3CR1^+^ cytotoxic effector phenotype and anti-tumor activity (84). T_H_1 polarized CD4^+^ T cells offer long-term protection against tumor re-challenge and is required for response to immune checkpoint blockade therapy in a T3 murine sarcoma model (85). Based on their study with melanoma patients who showed prevalence of CD4^+^ neoantigen-reactive T cells, Ott et al. suggested two mechanisms underlying this unexpected dominance of CD4^+^ over CD8^+^ T cells, namely: 1) more efficient priming of CD4^+^ T cells compared to CD8^+^ T cells due to restriction of cross-presentation and 2) relatively higher promiscuity of MHC class II epitopes owing to relaxed binding requirements, unlike MHC class I epitopes (86).

### Role of DC to Relay CD4^+^ ‘HELP’ to CD8^+^ T Cells

Priming of CD8^+^ T cells for effector function requires antigen cross-presentation with help from CD4^+^ T cells. The primary mechanism is *via* ‘licensing’ of DC that allows cross-presentation, essential for two-step priming of CD8^+^ T cells (2). To understand the spatiotemporal distribution and activation of CD4^+^ vs CD8^+^ T cells, *in vivo* imaging has demonstrated that after immunization, in the first priming step, CD4^+^ and CD8^+^ T cells encounter antigen in an independent and non-synchronous manner, presented by different subsets of cDC. Interaction between CD40 costimulatory protein on cDC and its cognate ligand CD40L (CD154) on CD4^+^ T cells is the key step in the licensing process that enhances antigen presentation on DC and allows direct interaction with CD8^+^ T cells.

The second step of priming these licensed type 1 cDC (cDC1) acts as a common platform where both CD4^+^ and CD8^+^ T cells encounter the same cDC1. XC-chemokine ligand 1 produced by CD8^+^ T cells recruits resident XC-chemokine receptor XCR1^+^ cDC in a prime position for receiving cross antigen presentation and thus, molecular help from CD4^+^ T cells is delivered to CD8^+^ T cells (2). Ahrends et al. demonstrated by RNAseq in ‘helped’ vs ‘non-helped’ CD8^+^ T cells that there is a differential expression of a multitude of genes associated with lymphocyte activation, differentiation, cell motility, and migration. Significantly enhanced mRNA and protein expression of cytotoxic effector molecules such as TNF-α, IFN-γ, FASL and granzyme B, as well as IL-2 and its receptor CD25, are regulators of CTL survival and memory. They also reported high levels of co-inhibitory immune receptors, e.g. PD-1, lymphocyte activate gene 3 (LAG3) and B and T lymphocyte attenuator (BTLA) on ‘helpless’ CTLs, rendering them unable to kill tumor cells even though they are able to exit the lymph node and enter circulation (87). These helpless T cells subsequently undergo activation-induced cell death due to TRAIL expression upon re-stimulation (88). In therapeutic pre-clinical models, vaccination with short MHC class I binding peptides hinders CTL priming and induce tolerance, whereas combination with CD40 agonist antibody infusion or DC stimulated *in vitro* with antigen-specific CD4^+^ T cell resulted in CTL-based anti-tumor immune response (2).

Secretion of CCL3 and CCL4 from the licensed DC guide the naïve CD8^+^ T cells to the site of antigen specific DC-CD4^+^ T cell interaction, that allows rapid interaction with the antigen presenting cDC1 even with a low frequency of both immune cell subtypes (89). CD4^+^ T cells also stimulate clonal expansion of antigen-specific CD8^+^ T cells and IFN-γ secretion, whereas ‘helpless’ memory CTLs primed without help from CD4^+^ T cells show deficiency in secondary expansion (90, 91). CD4^+^ T cells are a major source of IL-2, a key molecular help that is critical for imprinting the secondary responsiveness on CD8^+^ T cells. IL-15 is secreted from licensed DC and is considered to be necessary for imprinting secondary responsiveness even in absence of CD4^+^ T cells (92).

### CD4^+^ Help in CD8^+^ T Cell Differentiation and Memory Function

Recent research has highlighted that the impact of CD4^+^ T cell help reflects on enhanced recruitment, proliferation, and effector function of CD8^+^ T cells intratumorally. In a murine tumor model, IL-2 secreted from tumor-resident CD4^+^ T cells increased CD8^+^ T cell proliferation and granzyme B expression (93). Poor survival and clonal expansion of CD8^+^ T cells in absence of CD4^+^ help has been reported, and the help was necessary for survival of memory T cells during recall expansion (81). During clonal expansion and differentiation of T cells into short-lived effector or persistent memory phenotypes, CD4^+^ T cells help in intrinsic function of CD8^+^ T cells by altering gene expression profile. The transcriptomic analysis by Ahrends et al. also showed CD4^+^ T cell help induces transcription factors and epigenetic modulators, such as T-BET, eomesodermin homologue, and inhibitor of DNA binding 3, in a preventive model that received vaccines encoding MHC class I vs MHC class II-restricted epitope-expressing HPV E7 protein. Elevated expression of CXCR4 and CX3CR1 chemokine receptors and matrix metalloprotease proteins on ‘helped’ CTLs augment their extravasation and infiltration into the tumor (87). Another study using a similar mouse model showed CD4^+^ ‘help’ has been shown to impact transcriptional landscape to support formation and maintenance of CD8^+^ effector and central memory phenotypes, and recall response in these memory T cells were help-independent (94). Defective recall response mounted by CD8^+^ memory T cells from a CD4^-/-^ mouse host indicated necessity of CD4^+^ help for CD8^+^ T cell functionality previously (91), and a recent study using an Influenza A virus infection model showed CD4^+^ T cell help promotes metabolic programming of CD8^+^ T cells to benefit recall response as well (95).

### Molecular Nature of the ‘HELP’ Signal

#### Cytokine Signals

The key cytokine signals that deliver CD4^+^ T cell help to CD8^+^ T cells are IFN-γ and IL-12 secreted from conventional and CD40-stimultaed DC, respectively. It appears contribution of these two cytokines may work in a partially redundant manner, as they both promoted survival and differentiation of effector and memory CTLs by increased expression of transcriptional regulators in a mouse model (96). CD4^+^ T cells are a major source of IL-2, a key molecular signal that is critical for imprinting the secondary responsiveness on CD8^+^ T cells. IL-2 induces NAB2 protein expression by CD8^+^ T cells, inhibits TRAIL expression and promotes expansion (97). Simultaneously, IL-12p70 from licensed DC also upregulates IL-2Rα/CD25 expression on CD8^+^ T cells and therefore, enhances their responsivity to IL-2 (98). Our group reported a novel function of IL-12 to enhance recognition of tumor by T cells along with 10- to100-fold increases in peptide sensitivity and functional avidity (99). As reviewed by Kalia and Sarkar, IL-2 promotes differentiation into effector phenotype and contributes to the development and maintenance of short-lived effector responses by interaction with CD25 receptor (100). IL-15 is secreted from licensed DC and is considered to be necessary for imprinting secondary responsiveness even in absence of CD4^+^ T cells (92).

#### Co-Stimulatory Signals

Along with cytokines, costimulatory signaling between ligands and receptors expressed on DC, CD4^+^ and CD8^+^ T cells relay and implement CD4^+^ T cell help for T cell priming and effector function. Upregulated CD40L on CD4^+^ T cells interacts with its cognate receptor CD40 on DC and is the first step in relaying molecular help (4, 101, 102). Similarity of the cytokine profiles between CD4^+^ T cells and CD8^+^ T cells expressing CD40L has been reported and can potentially augment licensing of DC to enhance antigen cross-priming (103). CD70/CD27 costimulatory signaling has been reported to be the key mechanism to deliver CD4^+^ T cell help from DC to CD8^+^ T cells, and contribute to their clonal expansion and differentiation into effector and memory CTL in cancer and viral infections (104, 105). In a murine lung tumor model, CD27 agonism combined with anti-PD1 antibody treatment eradicated tumors and recapitulated CD4^+^ T cell help when vaccinated without helper epitopes (106), even though CD70/CD27 interaction alone may not stimulate sufficient CTL response and a combined, non-redundant role of CD27 and CD28 may contribute to the help. CD40-CD40L interaction stimulates CD80 and CD86 costimulatory molecule expression on DC and its subsequent binding with the CD28 costimulatory receptor on CD8^+^ T cells can deliver the CD4^+^ T cell help required for CTL activity, as observed in recent pre-clinical and clinical studies including anti-PD-1 and other immune checkpoint inhibitors (107, 108).

### Opposing Action of Anti- and Pro-Tumorigenic CD4^+^ T_H_ Cells in Cancer

**Figure 2:**
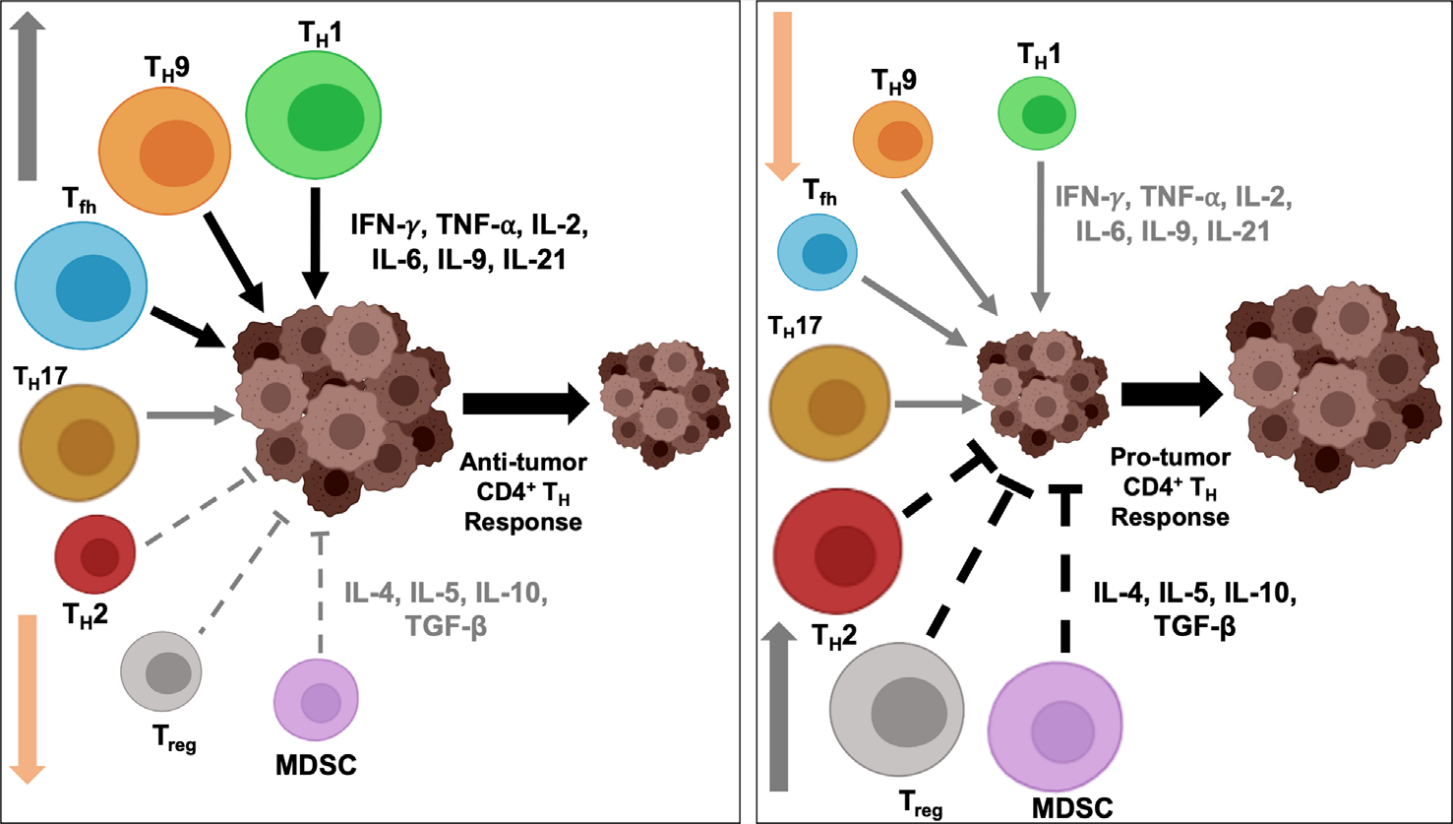
Intratumoral balance of anti- *vs* pro-tumorigenic CD4^+^ T_H_ cells determine immune response outcome. Activation and downregulation of specific T_H_ cells modulate intratumoral balance of stimulatory and suppressive effectors and modulates tumor response to immunotherapy. Polarization and activation of T_H_1, T_H_9 and T_fh_ subtypes induce secretion of proinflammatory cytokines, and, coupled with simultaneously diminished activity of immunosuppressive T_H_2 and T_reg_ cells, tip the balance towards anti-tumor immune response and induce tumor regression (*left)*. On the contrary, heightened activity of the immunosuppressive populations and secretion of inhibitory cytokines, and concurrent downregulation of immunostimulatory T_H_1, T_H_9 and T_fh_ cells induce a protumorigenic microenvironment, resulting in disease progression (*right*). To shift the balance to either end of the equilibrium, a concerted effort by multiple T_H_ subtypes are necessary and may not be achieved by alteration in the functional state of a single T_H_ subtype.

Research in past decades have revealed the critical and opposing role of T_H_1 and T_H_2 cells in determining the fate of intratumoral immune response, including therapies targeting oncodrivers and neoantigens. As shown in **Figure 2**, the regulatory network is multi-faceted and is governed by a range of cytokines and chemokines secreted from different T_H_ subtypes and hence, need to be coordinated in a balancing act for optimum efficiency of immunotherapy. We discuss the most well-known cytokines and chemokines secreted primarily from T_H_1 and T_H_2 cells that confer their anti- and pro-tumorigenic effects, respectively, to understand the mechanism of their opposing actions. The cytokines and chemokines secreted from the other T_H_ subsets have been summarized in **Table 1**. 

**Table 1:** Role of cytokines and chemokines in TH cell differentiation.

	T_H_1	T_H_2	T_H_9	T_H_17	T_FH_
**Differentiation Factors**	IL-2, IL-12, IFN-γ, IFN-α	GATA3, IL-4, IL-6	GATA3, IL-6, PU.1, TGF-β	TGF-β, IL-6, RORγt, STAT3	Bcl6, ICOS, IL-6, IL-21, STAT3
**Secreted Cytokines**	IL-1β, IL-2, IL-12, TNF-α, IFN-γ	IL-4, IL-5, IL-10, TGF-β	IL-9, IL-3, IL-21	IL-17A, IL-17F, IL-21, IL-22, IL-23	IL-21, IL-4
**Chemokines and cognate receptors**	CXCR3, CCR5, and CCR7, CXCL9, CXCL10, CXCL11,	CCR3, CCR4, and CCR8, MDC, TCA3, TARC	CCL20, CCR6,	CCL4, CCL17, CCL22	CXCR3, CCR6
**Classical Negative Regulatory Cytokines**	IL-4	IL-12	IFN-γ/IRF-1	IL-12, IFN-γ	IL-2
**Significant Downstream Signaling Pathways**	IRF1, STAT1, MAPK, STAT3	STAT5, STAT6	STAT6, IRF-4	STAT3	STAT3

### Anti-Tumorigenic T_H_ Cytokines

#### Interferon-γ (IFN-γ)

CD4^+^ T_H_1 effector cells are characterized by the production of dominant cytokines IFN-γ, TNF-α and IL-2. IFN-γ is a pleotropic cytokine and an important player in anti-tumor immunity with the ability to directly mediate tumor rejection as well as recruit and activate both innate and adaptive immune cells in the TME (109–112). The direct tumoricidal effects of IFN-γ result in cell death signaling and inhibition of angiogenesis. Increased expression of cell cycle regulators p21 and p27 induced by IFN-γ leads to cell cycle arrest, cell dormancy, and apoptosis in tumor cells *via* signaling pathways that induce the expression of tumor suppressor gene IRF-1, leading to caspase activation and apoptosis (109, 112, 113). Activation of the anti-proliferative STAT1 pathway by IFN-γ can lead to sensitization of tumor cells to FAS (CD95) and TRAIL resulting in apoptosis, and hindered tumor cell growth by inhibiting angiogenesis to induce a state of cellular dormancy (17, 109, 111–113). Our lab has recently elucidated a novel mechanism of IFN-γ action *via* ubiquitin proteasomal degradation pathway, mediated by zinc RING finger E3 ubiquitin ligase cullin-5, to facilitate proteasomal degradation of HER2 membrane receptor and improve response in HER2+ breast cancer *in vitro* and *in vivo* (114).

In the TME, IFN-γ enhances the immunogenicity of tumor cells by upregulating MHC class I and II expression to make them more susceptible to immune recognition (109, 111, 112) and influences the stromal cells in the TME including macrophages, myeloid-derived suppressor cells (MDSC), and DC (109). IFN-γ production leads to enhanced proinflammatory functions and tumoricidal activity of type I macrophages (M1) by increasing nitric oxide production and upregulates expression of MHC and costimulatory molecules on DC. Anti-tumor immune response by IFN-γ can also be elicited by recruitment of additional effector cells, namely NK cells and M1 macrophages to the TME, facilitating T-cell homing through CXCL9 and CXCL10 chemokines, and *via* enhanced CD8^+^ cytotoxicity in the TME (109, 111, 112).

#### Interleukin 2 (IL-2)

IL-2 plays a crucial role in driving T and NK cell proliferation and activation and in regulating their effector functions, such as cytolytic activity and cytokine production. IL-2 binds to IL-2 receptor (IL-2R), composed of three subunits: IL- 2Rα (CD25), IL-2Rβ (CD122), and IL-2Rγ (CD132) (115, 116). The heterotrimeric complex of IL-2αβγ is essential to regulate T cell expansion, are expressed on regulatory T cells, and binds IL-2 with the highest affinity, while T cells and NK cells express only the receptor dimer IL-2βγ (115–118). IL-2 is produced primarily by activated CD4^+^ T cells after antigen exposure, binds to its cognate receptors and drives differentiation to CD4^+^ T_reg_ immunosuppressive population that leads to immune tolerance. Research in the last decade has identified the role of IL-2 in promoting both T_H_1 and T_H_2 differentiation, and inhibiting T_H_17 and T_fh_ development (Liao et a, 2013). IL-2 induced activation of AKT and mTORC1 signaling pathways have been shown to steer the differentiation preference towards T_H_1 cells and away from T_fh_ subtypes (119). Binding of IL-2 to these receptor complexes induces signal transduction through three proliferative pathways: JAK/STAT, PI3K/AKT, and MAPK (115, 117, 118). Additionally, the positive feedback from CD4^+^ T_H_1 produced IL-2 plays a crucial role in driving T cell effector differentiation and in the recruitment of activated cytotoxic NK and CD8^+^ T cells to the TME (116). While IL-2 was the first FDA approved immunotherapy for metastatic melanoma and metastatic renal cancer, the dual functionality of IL-2 has been reported to regulate immunosuppressive environment intratumorally (115–117). Low concentrations of IL-2 have been shown to promote T_reg_ function in the TME, whereby anti-tumor therapies that employ high dose IL-2 attempt to overcome this immunosuppressive role (115, 117).

#### Tumor Necrosis Factor-α (TNF-α)

Tumor necrosis factor-α (TNF-α) is one of the primary proinflammatory cytokines produced by CD4^+^ T_H_1 cells and binds to two receptors, TNFR1 and TNFR2, that promote cell death and destruction of tumor vasculature. TNFR1 is expressed on various tumor and endothelial cells and is associated with pro-apoptotic signaling *via* MAPK and NFκB activation (111, 113, 120). Similar to the double-edged sword of IFN-γ, TNF-α has shown a dual tumor suppressing and tumor promoting role dependent on concentration and localization of the soluble cytokine. As TNFR1 is ubiquitously expressed on tumors as well as healthy endothelial cells and blood vessels, chronic exposure to TNF-α can cause non-specific tissue damage and has been linked to hemorrhagic necrosis (111). Additionally, TNFR2 is expressed primarily on immune cells including T_reg_ and MDSC (111, 120), where acute production of TNF-α is associated with T_reg_ expansion and increased infiltration of T_reg_ and MDSC populations in the TME, leading to tumor progression and decreased efficacy of immunotherapies (111, 121). Administration of even a low dose of TNF-α has shown increased expression of immunosuppressive molecules PDL-1 and TIM-3 on TIL and activate cell death pathways in tumor-infiltrating CD8^+^ CTL (121). Studies have shown that administration of TNF-α as an immunotherapy has resulted in high levels of toxicity, but localized delivery in isolated limb perfusion showed anti-tumor abilities in soft tissue sarcomas, melanoma, and hepatocellular carcinoma (111, 120).

### Anti-Tumorigenic T_H_ Chemokines

In addition to T_H_1 cytokines, the production of related chemokines has direct implications in shaping the immune landscape and TME of various cancer types. CD4^+^ T cell IFN-γ-inducible chemokines CXCL9, CXCL10, and CXCL11 recruit effector T cells to the TME, direct tumor infiltration, and are key players in T cell homing (122–125). CXCL9-11 bind to their cognate chemokine receptor CXCR3, which is expressed on cytotoxic CD8^+^ T cells, NK cells, and CD4^+^ T_H_1 cells (123, 124). Upregulation of CXCR3 on activated CD4^+^ T cells is associated with optimal production of IFN-γ and a T_H_1 effector phenotype (122). Additionally, CD40/CD40L signaling increases expression of CXCL10 and has been implicated in licensing DC and supporting the interactions of DC and naïve T cells in lymphoid organs (122, 126). CCL3 and CCL4 chemokines are released after interaction of DC with antigen specific CD4^+^ T cells and act as a chemoattractant for CCR5^+^ naïve CD8^+^ T cells for activation (89). Interaction of CCL19 and CCL21 with CCR7 receptor recruits T_regs_, CD4^+^ T_H_, T_CM_, and monocyte-derived dendritic cells (mDC) to the TME. Upregulation of CXCR3 and CXCR5 chemokine receptors has been correlated with T_H_1 differentiation while T_H_2 cells express CCR4 receptors, induced by IL-4, to bind CCL17 and CCL22 chemokines (125). Simultaneously, CXCL9 and CXCL10 have been shown to increase levels of tumor infiltrating CD8^+^ effector T cells and NK cells, minimize metastasis, and are correlated with improved responses to checkpoint blockade and adoptive cell transfer therapies (124, 125).

## Anti- vs Pro-Tumor T_H_ Immune Response in Cancer: Molecular Mechanism of Opposing Actions

T_H_1 and T_H_2 cells are often discussed in tandem in relation to cancer and tumor immune response, as T_H_1/T_H_2 balance, regulated by the factors summarized in **Table 1**, is paramount in tumor-specific immune response versus pro-tumor immune regulation. Typically, a shift in favor of T_H_1 response results in dissipated T_H_2 response and vice versa, resulting in either anti- or pro-tumorigenic consequences, respectively, and this shift is typically accomplished by antagonistic interaction of the cytokines produced by the T_H_ cells themselves (127). Depending on the TME and other external signals, the initial shift to either T_H_1 or T_H_2 can then become a positive feedback loop that continues to favor the specific T_H_ immune response (40).

T_H_1 and T_H_2 cells and their related cytokines have been studied in multiple cancer types and proved to play a pivotal role in prognosis, tumor fate, and patient disease-free survival. In one such study of patients with hepatocellular carcinoma, an increase in detectable IL-6 in whole blood after treatment with trans-arterial chemoembolization corresponded with a poorer prognosis and decreased overall survival rate. In the same study, a higher IFN-γ/IL-10 ratio increased overall survival rates, as did a higher IL-1/IL-10 ratio (128). In a similar study analyzing serum levels of cytokines in patients with invasive uterine cervical cancer, T_H_2, T_H_17, and T_reg_ cells were increased in peripheral blood mononuclear cells (PBMC) with a concurrent increase in their related cytokines IL-4, IL-10, IL-17, IL-23, and TGF-β (129). In a study using The Cancer Genome Atlas (TCGA) looking at glioblastoma multiforme, a low T_H_2 balance correlated with better overall survival (130). These studies exemplify the importance of maintaining a T_H_1-high/T_H_2-low balance and the ability to use relative T_H_ cell prevalence and their related cytokine levels as a predictor of patient survival. However, incidence of cancer is not necessarily indicative of a pro-tumor high ratio of T_H_2 over T_H_1, as shown in a study looking at patients with ovarian cancer before receiving treatment. When T_H_1 and T_H_2 cytokine levels in the serum and cancer tissues were compared, T_H_1 cytokines IL-2, TNF-α, IFN-γ, and IL-13 were significantly increased in patients compared to healthy controls. Additionally, IFN-γ/IL-4 and TNF-α/IL-4 ratios were significantly higher in cancer patients (131).

It has been previously shown that elevated T_H_2 cytokines (IL-4, IL-10) and decreased T_H_1 cytokines (IFN-γ, IL-2 and IL-12) correlate with poorer prognoses in breast cancer patients than those with elevated T_H_1 and suppressed T_H_2 cytokines. A recent study showed that alteration in T_H_1/T_H_2 cytokines can correlate with different subclasses of breast cancer as well. Shift in the T_H_1/T_H_2 balance towards a higher ratio of T_H_2/T_H_1 cytokines resulting in increased IL-4, IL-5, and IL-10 has been reported in TNBC. On the other hand, ER+ and luminal-like breast cancers were found to have lower T_H_2 cytokines and a general shift towards T_H_1 immune response. In the context of disease prognosis, such T_H_1/T_H_2 distribution is reflected in a better overall survival rate and prognosis in ER+ and luminal-like breast cancers (BC), and a worse prognosis in TNBC (132). T_H_1/T_H_2 balance in normal and cancer-associated immune response has been reviewed in general extensively elsewhere (29, 133). With respect to the cancer milieu, treatments that ensure a shift towards the anti-tumor T_H_1 response are essential, while maintaining a low T_H_2 response is critical to ensure a tumor-specific immune response is maintained.

In the context of pro-tumorigenic immune response in the TME, T_H_2 response has been viewed as controversial, due to their possible role in tumorigenesis, along with another CD4^+^ T_H_ cell subset, T_H_17 cells. T_H_2 cells are responsible for the increase in population of tumor infiltrating M2 macrophages and eosinophils in the TME, *via* their expression of IL-5 and IL-13, which regulate TGF-β secretion and immunosuppressive responses (28). T_H_2-induced tumorigenesis is further driven by their expression of IL-7, which can act as a pro-angiogenic factor, resulting in leaky vasculature and allowing the tumor microenvironment to expand and migratory tumor cells to enter the surrounding tissue (134). A study involving luminal breast cancer found that the presence of chemokine receptor CCR5 activates T_H_2 differentiation, and the T_H_2 cells in turn, help increase MDSC production within the TME, a characteristic feature shared by T_H_17 cells as well but implemented *via* IL-17 secretion. A large enough population of MDSCmigrates to the edge of the tumor in order to prevent TIL from entering the tumor region and can severely diminish the immune response, allowing the tumor to thrive (135). A comprehensive understanding of how these T_H_ cells induce pro-tumorigenic immune response requires further research, to identify efficient strategies to repress the immunosuppressive populations and expand therapeutic benefit of T_H_ cell-based immunotherapy in cancer.

## Regulation of B Cells by CD4^+^ T Cells: a Bidirectional System

Discovery of tumor infiltrating B cells (TIB) and tertiary lymphoid structures have reinforced interest in studying the significance of TIB subtypes and success of immunotherapy in cancer. Such studies have identified a dual role of TIB subtypes in stimulating or dampening of anti-tumor immune response, orchestrated by secreted antibodies, cytokines and chemokines, B cell receptor signaling, and interaction with T cells. CD4^+^ T cells act in a bidirectional regulatory network with B cells to induce differentiation of B cells which in turn, stimulates CD4^+^ T_H_1 and T_H_2 differentiation, suggesting the clinical significance of these immune cells for anti-tumor response. Similar to the conventional APC, B cells express MHC class II molecules on their surface and are, hence, capable of antigen presentation to CD4^+^ T cells for activation, and cognate interaction between T and B cells induce differentiation of anti-tumor T_fh_ cells (49, 136). Activated B cells secrete chemokines and costimulatory factors such as CCL2, CXCR4, CCL5, CXCL5, and CXCL10 to induce CD4^+^ and CD8^+^ T cell activation. Using B cell deficient and IFN-γ knockout mice along with other transgenic models of immune cell function and CD4^+^ and CD8^+^ depletion studies, Park et al. showed that anti-HER2/neu antibodies are necessary and sufficient for protection from tumor challenge, a temporary necessity for CD4^+^ T cells for 36-48h after immunization to provide help for B cells, and no requirement for CD8^+^ T cells at all. While tumor growth in immunized B cell-deficient mice was comparable to controls and showed no detectable antibodies in their serum, treatment of mice with anti-HER2 serum prevented tumor growth *in vivo* as effectively as adenoviral vaccination, supporting the necessity and sufficiency of antibodies for anti-tumor protection (137). In a later study, Berzofsky’s group demonstrated that even in a large and well-established subcutaneous TUBO tumor model (tumor size >2cm), vaccination with a recombinant adenovirus expressing a truncated ErbB2 antigen cured primary tumors and distant lung metastases in mice by antibody-mediated blockade of HER2/neu activity, in an Fc receptor-independent mechanism. Adoptive transfer of serum from vaccinated BALB/c mice to TUBO tumor-bearing mice resulted in significantly delayed tumor growth and showed considerable presence of anti-HER2/neu antibodies which were not observed upon deletion of CD4^+^ T cells (138), reinforcing the therapeutic benefits of anti-oncodriver antibodies and significance of CD4^+^ T cells.

On the other hand, inhibition of CTL activity in tumors by B cells can be associated with a subset of B regulatory cells (B_reg_), that contribute to immunosuppression in the TME. B_reg_ inhibit proliferation of CD4^+^ T_H_1 cells by secretion of suppressive cytokines, such as IL-10 and TGF-β, and promote conversion of CD4^+^CD25^−^ T cells to CD4^+^CD25^+^FoxP3^+^ T_reg_ with high expression of CTLA-4 and FoxP3, and the anti-tumor effects of B cell deficiency has been shown to be mediated by enhanced T cell and NK cell infiltration, vigorous T_H_1 and CTL activity and reduced T_reg_ proliferation (139). These studies underline the relevance of B cell mediated antibody production and CD4^+^ T cell activation in anti-tumor immune response to encourage further research in understanding the complete therapeutic potential of B and T cell interaction.

## Oncodriver-Specific and Neoantigen-Driven CD4^+^ T Helper Immune Response

### Oncodrivers in Cancer

Dependence of cancer cells on oncodrivers present them as a promising candidate for targeted therapy development since these proteins are critical for survival and malignancy of tumor cells and are often overexpressed in tumors compared to healthy cells. While oncodrivers are sufficient and/or necessary for normal physiological cellular functions, their overexpression and hyperactivity often become the key regulator of tumor proliferation and escape from cellular death. Perhaps the most prominent oncodriver investigated in the context of breast cancer is human epidermal growth factor receptor 2/receptor tyrosine-protein kinase erbB-2 (HER2/Erbb2), and other members of the ERBB family of receptors, namely EGFR/HER1, HER3 and HER4, have been established as potent oncodrivers in breast cancer, along with lung, ovarian, gastric and bladder carcinoma (140–142). HER2 status has been correlated with poor recurrence-free survival and disease-specific survival in ER^+^/HER2^+^ BC (143–145), resectable gastric cancer (146, 147), and pancreatic cancer (148). Enhanced HER2-HER3 interaction and HER3 activity in breast cancer cells provide an escape route for HER2^+^ cancer cells to switch dependence, continue PI3K/AKT activity and induce trastuzumab resistance (149). In ER^+^ BC, HER3 emerges as a potent inducer of tamoxifen resistance (150), and as a prognostic marker, HER3 expression has been associated with poor survival in TNBC and HER2^+^ BC (151–153). In a cohort of 510 TNBC patients, immunohistochemistry and RNA sequencing revealed that the combined HER3-EGFR score is a more comprehensive prognostic marker than individual HER3 and EGFR scores and high HER3-EGFR score predicts worse breast cancer-specific and distant metastasis-free survival, suppressed apoptosis-inducer ATM activity, activation of EGFR, PARP1, and caspases, and inhibition of p53 and NFκB (154). Significance and current status of oncodriver-targeted immunotherapy have been reviewed previously (155).

### Oncodriver-Specific T_H_1 Immune Response in Breast Cancer: HER2-DC1 Vaccine

Interaction between oncodrivers and immune response has been documented in HER2^+^ BC, where trastuzumab induces antibody-dependent cell-mediated cytotoxicity (ADCC) by facilitating cross-link between tumor antigen with its antigen-binding fragment and recruitment of effector cells by interaction with the Fc region (fragment crystallizable region), resulting in cytokine release and cytotoxic cell death (156). Susceptibility to ADCC correlates with infiltration of CD16 and CD56-expressing lymphocytes in the tumor, suggesting recruitment of NK cells (157). Trastuzumab has also been shown to stimulate HER2 uptake by DC for enhanced antigen presentation and activation of antigen-specific T cells (158). Higher levels of chemokines, infiltration of T cells and monocytes, and PD-1 expression has been documented on trastuzumab sensitive breast tumors, compared to non-responding tumors (159). Our lab has reported a gradual and progressive loss of HER2-specific CD4^+^ T_H_1 immune response in peripheral blood in HER2^+^ BC patients (160). Restoration of this T_H_1 immune response with neoadjuvant HER2 peptide-pulsed type I DC (HER2-DC1) vaccination resulted in pathologic complete response in 30% of HER2^+^ DCIS patients in a randomized trial (161). Co-operation between CD4^+^ T_H_1 cytokines IFN-γ/TNF-α and trastuzumab has been shown to be necessary for restoration of class I MHC molecule expression on HER2^high^ cells, critical for recognition and lysis of the cells by HER2-specific CD8+ T cells in these patients (162). In HER2^+^ IBC completely treated with trastuzumab and chemotherapy, anti-HER2 T_H_1 immune responsivity independently correlates with disease recurrence and is mediated by anti-HER2 CD4^+^T-bet^+^IFN-γ^+^ (T_H_1) phenotypes but not CD4^+^GATA-3^+^IFN-γ^+^ (T_H_2) or CD4^+^CD25^+^FoxP3^+^ (T_reg_) response (163). At the cellular level, T_H_1 cytokine treatment up-regulated apoptosis and senescence in HER2^+^ BC cells (164), suggesting molecular communication between immune and oncodriver signaling. In a pre-clinical model of HER2^+^ BC, sequential anti-PD1 antibody treatment with murine HER2-DC1 vaccination significantly improves mouse survival and supports an essential role of CD4^+^ T_H_1 immune response for the observed effect (165). Therapeutic success of HER2-DC1 vaccination in HER2^+^ BC supports the notion that targeting other oncodrivers employing the DC vaccine platform can have far-reaching beneficial effects in breast and other cancers dependent on oncodrivers, such as TNBC, which otherwise lacks effective therapeutic strategy. HER3 deserves attention in this context as our lab has reported progressive loss of HER3-specific T_H_1 immune response in TNBC patients, and patients with residual or recurrent disease showed significant suppression of immune response compared to patients without recurrence or complete response after neoadjuvant chemotherapy (166). Future research will be essential for a comprehensive understanding of the interaction between oncodrivers and immune cell signaling in tumors, for developing efficient targeted immunotherapies with improved therapeutic success.

### Neoantigens and Neoantigen-Driven Immune Response

**Figure 3:**
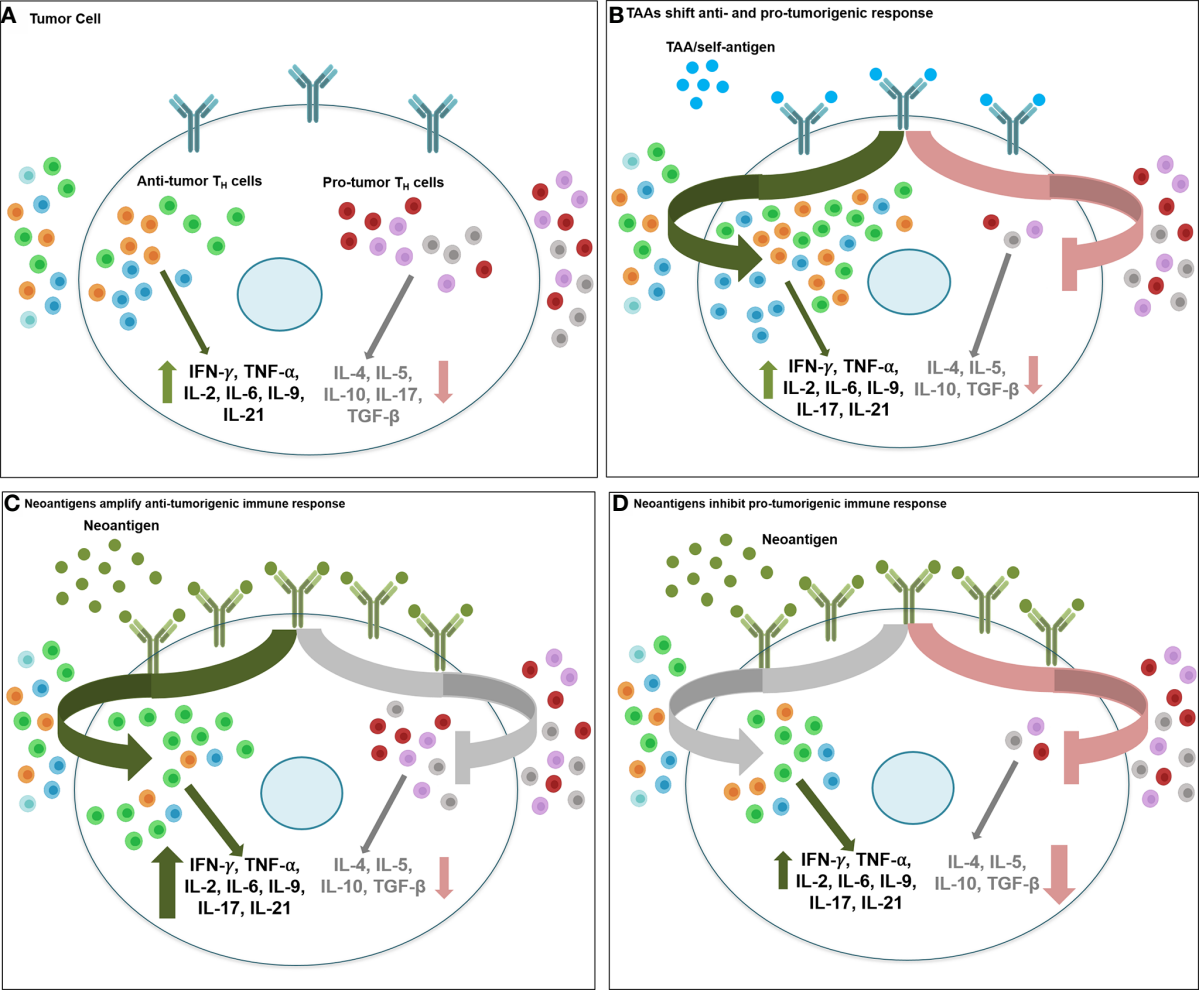
Therapeutic targeting of tumor associated antigens and neoantigens activate anti- vs. protumorigenic CD4+ T_H_ cell subtypes. Therapeutic targeting of oncodrivers/TAA/self-antigens may stimulate tumor immune response differently than strategies involving neoantigens. **(A)** Intratumoral balance of anti- and protumorigenic CD4^+^ T_H_ immune cell population maintain the equilibrium of inflammatory (IFN-γ, TNF-α, IL-2, IL-6, IL-9, IL-21) and inhibitory cytokines (IL-4, IL-5, IL-10, TGF-β) in cancer cell and determine overall immune response to therapy. **(B)** When tumor cells express self-antigens/TAA/oncodrivers (*blue spheres)*, due to low abundance and weak immunogenicity of these antigens, effective immunotherapy targeting these proteins may require a more extensive shift in the balance of anti- vs pro-tumor immune effector populations, including recruitment and activation of all anti-tumorigenic T_H_1/T_H_9/T_fh_ populations (*green arrow*) and suppression of all pro-tumorigenic T_H_2/T_H_17/T_reg_/MDSC function (*red arrow)*. Conversely, highly antigenic and abundant neoantigens (*green spheres)* may be sufficient to stimulate anti-tumor immune response either by **(C)** driving up infiltration and hyperactivation of primarilyT_H_1, along with T_H_9 and T_fh_ immunostimulatory response (*green arrow*) with minimal changes in the inhibitory immune cell function (*grey* arrow) or by **(D)** drastic downregulation of immunosuppressive response by T_H_2/T_H_17/T_reg/_MDSC cells (*red* arrow) without a significant change in the immunostimulatory population of T_H_1/T_H_9/T_fh_ cells (*grey arrow) (light green, T_H_1; red, T_H_2; orange, T_H_9; blue, T_fh_; dark green, T_H_17; grey, T_reg_; purple, MDSC)*.

While T cell activity towards tumor-derived neoantigen has been reported in mouse models as early as in the 1980s, they have gained renewed interest in recent years as significance of neoantigens to enhance ‘foreignness’ of tumors has been shown to be critical for success of immunotherapy, including immune checkpoint blockade therapeutics. Neoantigens are distinct from the tumor-associated antigens (TAA) which are proteins present in normal tissues and overexpressed in tumors, and therefore, peptides of TAA can be recognized by T cells following interaction with human leukocyte antigen (HLA). The most prominent TAA are HER2, MAGE, MUC1, NY-ESO-1, MART-1and mammaglobin-A, among others. Neoantigens, on the other hand, are unique non-autologous proteins expressed in tumor, due to somatic DNA alterations such as non-synonymous point mutations, insertion/deletion, gene fusion and frameshift mutations (167). Compared to TAA, neoantigens present a more appealing target for targeted immunotherapy development due to their higher immunogenicity that is enhanced because of increasing difference between the mutated and normal peptide sequence, strong individual tumor specificity, higher affinity towards MHC, and reduced risk of autoimmunity as they are recognized as foreign antigens and not affected by central immunological tolerance (168). Targeting TAA of low abundance and weak immunogenicity versus neoantigens that are abundant and highly immunogenic may not alter the intratumoral balance of anti- and pro-tumorigenic CD4^+^ T_H_1 immune response, and the overall success of immunotherapy, to the same extent. As shown in **Figure 3**, low antigenic load presented by oncodrivers/self-antigen/TAA may require a more comprehensive shift in the balance, including both activation of anti-tumorigenic T_H_1/T_H_9/T_fh_ response and suppression of pro-tumorigenic T_H_2/T_H_17/T_reg/_MDSC function, for effective immunotherapy; while the high abundance and immunogenicity of neoantigens may be enough to drive up one side of the balance, by either hyperactivating anti-tumorigenic response or severe suppression of immunoinhibitory populations in favor of anti-tumor immune response to result in superior therapeutic efficacy.

A series of studies have demonstrated correlation between tumor mutational burden and/or predicted neoantigen ‘load’ (abundance of neoantigens) and patient survival. Reports of a positive association between higher predicted neoantigen load and increased intratumoral lymphocyte infiltration (CD3^+^ and CD8^+^ T cells) and improved overall survival in colorectal, endometrial and ovarian cancer (169–171) led to studies addressing the relationship between neoantigen abundance and success of immune checkpoint blockade therapy in cancer. Indeed, in melanoma patients treated with anti-CTLA4 antibody, NSCLC patients receiving anti-PD1 antibodies and urothelial carcinoma patients receiving anti-PD-L1 therapy, the extent of DNA damage (that corresponds to tumor mutational burden and neoantigen load) correlates with therapeutic response (172–174). Even though a large number of studies have focused on teasing out the role of neoantigen-targeted CD8^+^ T cell activity in cancer, significant and preferential CD4^+^ T cell activation by neoantigens have been recognized in multiple pre-clinical and clinical studies (175). Current pre-clinical and clinical trials employ multiple platforms of neoantigen-targeted vaccines such as synthetic long peptide vaccine, DNA and RNA vaccine, and DC vaccine, along with adoptive T cell therapy. As reviewed previously, pre-clinical studies have demonstrated significant therapeutic benefit of neoantigen-targeted vaccines (167, 168). In three murine models of melanoma (B16F10), breast (4T1) and colon (CT26) cancer, the majority of the mutated neo-epitopes were recognized by CD4^+^ T cells, and vaccination with such mutations elicit robust tumor rejection (176). A recent study published with 4T1 and B16F10 murine models tested therapeutic efficacy of a novel cryo-thermal therapy with respect to conventional radiofrequency ablation and showed strong neoantigen-specific CD4^+^ T-cell response induced by cryo-thermal therapy, resulting in anti-tumor immune response and long-lasting protection against tumor re-challenge (177). Combination of local radiotherapy with an RNA-LPX vaccine that encodes CD4^+^ T cell-recognized neoantigens resulted in a poly-antigenic, potent CD8^+^ T cell response and memory that rejected CT26 tumor re-challenge, had higher number of polyfunctional IFN-γ^+^ CD4^+^ T_H_1 cells specific for the immunodominant CD4 neoantigen ME1, elevated numbers of activated gp70-specific CD8^+^ T cells, and lower PD-1/LAG-3 expression. Follow-up immunotherapy with anti-CTLA4 antibody resulted in complete remission of gp70-negative CT26 tumors in all mice in this study (178). In an inducible lung adenocarcinoma mouse model, vaccination using the G12D KRAS mutations as neoantigens and a novel synthetic long peptide-containing cationic lipoplex-based delivery platform stimulated both CD4^+^ and CD8^+^ T cell response and suppressed tumor growth, while combination with checkpoint inhibitor furthered such suppression (179). Similarly, both CD4^+^ and CD8^+^ T cell response has been reported in recent clinical studies following neoantigen-specific vaccination, across multiple cancer types. Whole-exome sequencing demonstrated that TIL in metastatic cholangiocarcinoma contained CD4^+^ T_H_1 cells specifically responsive against a mutation in ERBB2 interacting protein (ERBB2IP) and adoptive transfer of TIL containing mutation specific polyfunctional T_H_1 cells resulted in a decrease in target lesions with prolonged survival (180). CD4^+^ T cells capable of recognizing the recurrent KRASG12V and the ERBB2 internal tandem duplication oncodriver mutations were identified in PBMC samples collected from a small cohort of NSCLC patients (181). Frequent recognition of neoantigens by CD4^+^ T_H_1 cells have been reported in melanoma as well (182). In a phase I/Ib study reported last year, personalized neoantigen vaccination in glioblastoma patients increased tumor infiltrating cells, accompanied by a circulating polyfunctional neoantigen-specific CD4^+^ and CD8^+^ T cell responses enriched in memory phenotype, in patients who did not received dexamethasone (183). In treatment-naïve epithelial ovarian cancer patients, whole-exome and transcriptome sequencing analysis to identify neoantigen candidates and vaccination thereafter showed spontaneous CD4^+^ and CD8^+^ T-cell responses against neoepitopes from autologous lymphocytes in 50% of the patients, along with enhanced antigen processing and presentation machinery present in those specific tumors (184).

Therefore, along with further optimization of neoantigen prediction algorithm and targeting, a comprehensive understanding of the neoantigen recognition by CD4^+^ T cells and how that stimulates intratumoral effector and helper function of these T cells will be of utmost importance for the development of personalized immunotherapy targeting individual tumor neoantigens and demands extensive research.

## Immune Checkpoint Modulators and T_H_ Cell Regulation

The tumor microenvironment weighs heavily on T cell differentiation. An intratumoral meshwork of regulatory immune cells and immunosuppressive cytokines/chemokines act as one of the central modulators of T cell differentiation and function. TGF-β produced by tumors can convert CD4^+^ T cells into T_reg_ cells *in situ* (185). Recruitment of MDSC in the TME aid in this suppression of T_H_ immune cells, where TNF-α, IL-1, IL-6, colony stimulating factor 1 (CSF-1), IL-8, IL-10, and type I interferons can also play a role in the regulation of T_H_ immune response to tumor cells. VEGF, IL-10, and TGF-β have been shown to inhibit DC maturation, leading to poor antigen presentation and co-stimulation of T cells, which favors T_H_2 differentiation and shifts the balance from T_H_1 to T_H_2 phenotype (185). Manipulation of the TME, immune checkpoint regulation and cytokine levels may ensure long term tumor free survival in patients. Immune checkpoint blockades with anti-PD-1, anti-PDL1, and anti-CTLA4 antibodies to combat the inhibitory effects of TME on the immune system have been studied and showed promising therapeutic efficacy (186). Immune checkpoints are the gatekeepers of immune response and has garnered significant attention in the field of cancer immunosurveillance and immunotherapy in recent years. These inhibitory receptors/co-stimulatory molecules target T cell receptor (TCR) signaling activation, induce T cell exhaustion and anergy, and suppress proinflammatory cytokines (e.g. IFN-γ, TNF-α) secretion, ultimately resulting in immunosuppression in the TME and has been targeted with antibody-mediated checkpoint blockade therapy in recent years, as discussed comprehensively in recent reviews (187–189). Therefore, expression of these checkpoint regulators on T_H_1 as well as cytotoxic T cells, while negatively impacting their proliferation and function, can be critical in determining success of checkpoint blockade therapy in high versus low density immune checkpoint-bearing tumors. In classic Hodgkin’s Lymphoma, where MHC-I expression is lost but MHC-II expression is intact, CD4^+^ T cell infiltration in tumor was correlated with better prognosis in patients and showed improved efficacy of a PD-1 blocking antibody in MHC-II-expressing lymphoma. In the same study, Nagasaki et al. showed that CD4^+^ T cell cytotoxicity played a critical role in delivering anti-tumor effects of anti-PD1 antibody which was observed in MHC-I^−^MHC-II^+^ tumors, but not on MHC-I^−^MHC-II^−^ tumors, in murine models of lymphoma and solid tumors (190). Kagamu et al. investigated NSCLC patients receiving nivolumab immunotherapy and found that treatment responders had higher circulating level of effector, CD62L^low^ CD4^+^ T cells prior to PD-1 blockade that correlated with effector CD8^+^ T cell abundance, and these cells expressed surface markers indicative of T_H_1 phenotype (191). In a study with healthy subjects and glioblastoma multiforme (GBM) patients, PD1^+^ CD4^+^ T cells were found to be unable to proliferate but secrete IFN-γ and display exhaustion markers in RNA sequencing analyses. In GBM samples, enrichment of both PD1^+^ CD4^+^ and PD1^+^ TIM3^+^ CD4^+^ T cells suggest combined blockade of multiple checkpoints can be a requirement to tackle aggressive cancers like GBM (192). Varying levels of PD-1 expression on follicular lymphoma cells reflect on the T_fh_ phenotype of intratumoral CD4^+^PD-1^high^ T cells with no TIM3 expression that supports B cell growth, while CD4^+^PD-1^low^ T cells elicit an exhausted phenotype, express TIM3 with reduced cytokine secretion and cellular signaling, and significantly correlate with a reduced overall survival in follicular lymphoma patients (193). Another checkpoint modulator, CTLA-4, is constitutively expressed on CD4^+^CD25^+^ T_reg_ cells leading to trans-endocytosis of B7 ligands and interference with the CD28 co-stimulatory signaling, and has been deemed necessary for secretion of anti-inflammatory cytokines by T_reg_ cells (194, 195). Future research will be crucial to elaborate immune checkpoint regulation of CD4^+^ T_H_ cell differentiation and function and identify new nodes in the network for therapeutic targeting in cancer.

## T_H_ Immune Cells in Cancer Cell Dissemination, Dormancy and Metastasis

It is widely accepted that cancer cells disseminate from non-invasive or primary tumor sites into the circulation and reach various distant organs to form overt metastasis (196). T_H_1 cytokines such as IFN-γ, TNF-α and IL-2 produced by T_H_1 cells contribute to inhibit tumor growth and activation of tumor-specific immune mechanisms (155, 197). On the other hand, T_H_2 cytokines IL-10, IL-4 and TGF-β from T_H_2 cells can promote dissemination of cancer cells dissemination and metastasis in various cancers (198). The imbalance between the ratio of T_H_1/T_H_2 cells and their associated cytokines correlates with decreased progression-free survival and overall survival in patients with breast, melanoma, ovarian, esophageal and colon cancers (199). Previous studies in breast cancer patients have shown that presence of cancer cells in the systemic circulation are associated with alteration of CD4^+^ T_H_ cells (200, 201). After dissemination, cancer cells can remain dormant for a prolonged period until they emerge for metastatic colonization in secondary organs (202, 203). T_H_1 cells can reduce proliferation and mediate dormancy in these disseminated cancer cells (DCC) *via* IFN-γ dependent STAT1 signaling pathway activation and anti-tumor immunity (113). A mouse model of melanoma showed presence of DCC in various organs, such as the lungs, skin and reproductive tract, and regulation of their non-proliferative status by T_H_1 immune cells (204). Another study using a mouse model for sarcoma also supports a role of CD4^+^ T cells to induce dormancy in cancer cells and tumor relapse (205). These reports suggest the regulatory role of T_H_ immune cells in controlling tumor dormancy and metastasis. Since maintaining T_H_1/T_H_2 immune cell balance is critical in anti-tumor immunity, therapeutics that enhance T_H_1 response and prevent T_H_2 activation and associated cytokines may simultaneously help to eradicate disseminated cancer cells, preventing recurrence and metastasis.

Tumor infiltrating T_H_9 and T_H_17 cells are observed to promote epithelial to mesenchymal transition (EMT) and migration potential of lung cancer cells and metastasis outgrowth. IL-9 and IL-17 cytokines from T_H_9 and T_H_17 cells can stimulate cytokine signaling and alter various genes linked to EMT and drive metastasis (39). In addition, high accumulation of T_H_9 and T_H_17 cells in lung cancer patients with poor survival further support their multifaceted role in cancer progression and metastasis (39). Another study has demonstrated high serum level of IL-9 and IL-17 cytokines with increased frequency of T_H_9 and T_H_17 cells in hepatic carcinoma patients with malignant ascites (206). This finding suggests that T_H_9 and T_H_17 cells may play a significant role in metastatic spread through IL-9 cytokine signaling.

## Clinical Experience with CD4^+^ T_H_ Cells: Current Status

**Figure 4:**
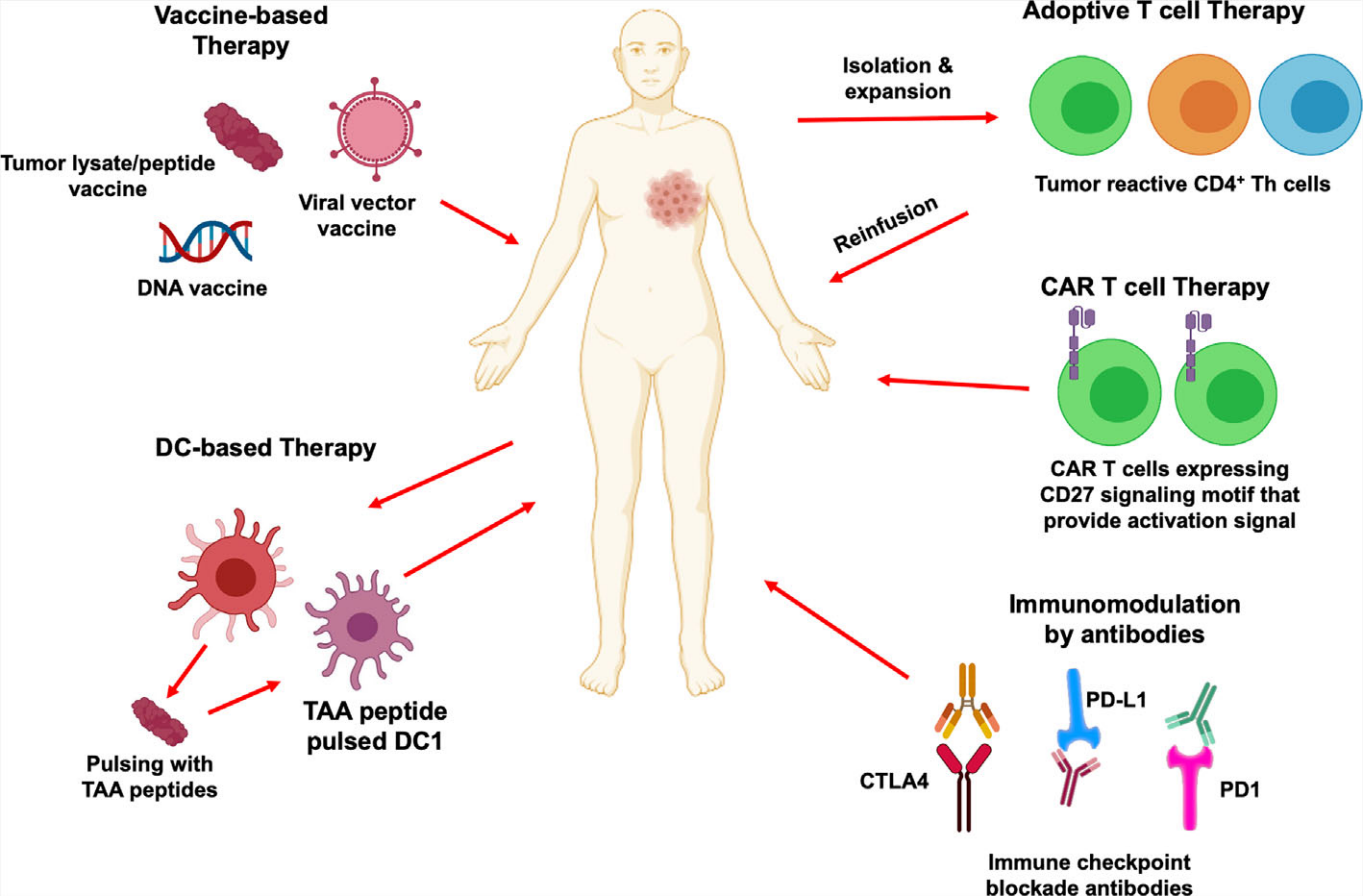
CD4^+^ T cells in cancer immunotherapy. Immunotherapeutic strategies that activate CD4^+^ T cells and their downstream effector immune cells for cancer treatment are depicted. Therapeutic vaccination includes tumor antigenic peptides, viral vector-based vaccine and DNA based vaccine that can mediate CD4^+^ T cells immune responses. DC-based vaccines can prime CD4^+^ T cells and create signals to activate cytotoxic CD8^+^ T cells differentiation and anti-tumor function. Adoptive transfer of tumor specific CD4^+^ T cells is another attractive immunotherapy approach which helps to develop specific and strong anti-tumor immune reaction. Chimeric antigen receptors can also redirect CD4^+^ T cells and provide activation signals to recognize cancer cells to eliminate them. Blockade of immune checkpoints PD1, PD-L1 and CTLA4 by antibodies can prevent tumor associated immunosuppressive environment and enhance tumor specific CD4^+^ and CD8^+^ T cells immune responses.

In the past decade, it has become clear that CD4^+^ T cells play a multifaceted role and are crucial for generating effective anti-tumor immunity. Therapeutic approaches designed to target CD4^+^ T cell responses can be broadly divided into passive immunotherapy (antibody-based therapies, adoptive cell therapy, and chimeric antigen receptor T cell therapy) and active immunotherapy (peptide vaccines, DC-based immunotherapies, immune checkpoint blockade). Here, we review current status of these immunotherapeutic approaches to stimulate tumor specific CD4^+^ T cell responses, focusing on peptide vaccines, adoptive T cell transfer and chimeric antigen receptor T cell therapy (**Figure 4**). Ongoing clinical trials utilizing these immunotherapy strategies have been summarized in **Table 2**.

**Table 2:** Clinical trials evaluating the role of TH Cells in response to immunotherapies.

ClinicalTrials.gov Identifier	Intervention	Target patients	Immune Outcome measure	Primary endpoint
NCT03946358 Phase II	Atezolizumab (anti PD-L1) and UCPVax (vaccine), Blood sample collection, Tumor biopsies, CT scan	Squamous Cell Carcinoma of the Head and Neck Anal Canal Cancer Cervical Cancer		Objective response rate at 4 months
NCT03904537 Phase I/II	anti-PD-1 antibody-activated TILs	Colorectal Cancer Stage III	CD3+, CD8+, CD4+ or CD56+ T cells	6 months
NCT03844763 Phase I/II	Avelumab (anti PD-L1), Radiation, and CTX (cyclophosphamide)	Head and Neck Cancer		Objective response rate (2-4 months)
NCT03734692 Phase I/II	Cisplatin, Pembrolizumab, Rintatolimod	Ovarian Cancer Recurrent	Pre-and post-treatment CD3+, CD4 Tbet+, CD8+, NK cells and granzyme B	13 weeks
NCT03698461 Phase II	Atezolizumab, Bevacizumab, Oxaliplatin, Levoleucovorin, 5-fluorouracil	Colorectal NeoplasmsNeoplasm MetastasisColonic NeoplasmsRectal Neoplasms	CD3, CD4, CD8 T cells, PD-L1, PD-1, CD45RO, FOXP3, CD68, Granzyme B	End of treatment
NCT03410732 Phase II	activated DCs, radical surgery only	Gastric Cancer	CD4/CD8 T cell percentage change	Progression free survival (3 years)
NCT03067155 Phase II	CMV-specific T cells, Standard anti-viral therapy	Hematological Malignancies CMV Infection		1 year
NCT02818426 Phase I/II	UCPVax (peptide vaccine)	Metastatic Non-small Cell Lung Cancer		57 days (phase I) 73 days (phase II)
NCT02957968 Phase II	Doxorubicin, Cyclophosphamide, Paclitaxel, Carboplatin, Decitabine, Pembrolizumab	Breast Adenocarcinoma Estrogen Receptor- Negative Breast Cancer Estrogen Receptor-positive Breast Cancer HER2/Neu Negative Invasive Breast Carcinoma Progesterone Receptor Negative Progesterone Receptor Positive Tumor Stage IIA Breast Cancer Stage IIB Breast Cancer Stage IIIA Breast Cancer Stage IIIB Breast Cancer Triple-negative Breast Carcinoma	TIL %, CD3, CD4, CD8 T cells, Treg, MDSC, B cell, PD-1, PDL-1	29 days
NCT01868490 Phase I/II	cytokine induced killer cells	Cholangiocarcinoma		6 weeks
NCT03384914 Phase II	WOKVAC Vaccine, DC1 Vaccine	HER-2 Positive Breast Cancer	Immunogenicity (IFN-γ ELISPOT)	Up to 7 years
NCT04552886 Phase I	TH-1 Dendritic Cell Immunotherapy	Glioblastoma		2 years
NCT04157127 Phase I	Autologous DC vaccine	Pancreatic Adenocarcinoma Pancreatic Cancer		6 weeks
NCT02846103	blood and tumor tissue samples (Immune monitoring)	Lung Cancer	UCP-specific Th1 response (IFN-γ ELISPOT)	2 years
NCT03387553 Phase I	Dendritic Cell Vaccine (DC1), Neoadjuvant Chemotherapy, Curative Surgery	HER2-positive Breast Cancer	HER2-specific Th1 response (IFN-γ ELISPOT)	28 weeks
NCT03977103 Phase II	High dose irradiation conditioning + Treg/Tcon	Acute Myeloid Leukemia Acute Lymphoid Leukemia Myeloproliferative Disorders Lymphoma Multiple Myeloma Other Hematologic Malignant Neoplasms		2 years
NCT03696030 Phase I	Chimeric Antigen Receptor T-Cell Therapy	Metastatic Malignant Neoplasm in the Leptomeninges Breast Cancer HER2-positive	Myeloid, B cells, T cells (including subtypes) in CSF, blood and TME, cytokines in CSF and blood	21 days up to 15 years
NCT04433221 Phase I/II	Multiple sarcoma-specific CAR-T cells and sarcoma vaccines	Sarcoma Osteoid Sarcoma Ewing Sarcoma		3 months
NCT01955460 Phase I	Aldesleukin (Recombinant Human IL-2) Cyclophosphamide Fludarabine Phosphate NGFR-transduced Autologous T Lymphocytes TGFbDNRII-transduced Autologous Tumor Infiltrating Lymphocytes Laboratory Biomarker Analysis	Metastatic Melanoma		Up to 5 years
NCT03112590 Phase I/II	Interferon-gamma (IFN-γ) Paclitaxel Trastuzumab Pertuzumab Post Therapy Surgery	HER2-positive Breast Cancer		End of treatment

### Vaccines

Cancer vaccines were developed to stimulate specific anti-tumor T cell responses by (1) developing antigen-loaded DC *ex vivo* prior to vaccination (DC vaccines) and/or (2) directly administering immunogenic peptides (epitope-based vaccines). Antigens used in vaccination may span from use of peptide fragments or full proteins, DNA and mRNA, or even bulk cancer cell lysates to stimulate CD4^+^ T_H_1 responses *in vivo* (207, 208). The first efforts to active CD4^+^ T_H_1 anti-tumor immunity were actually implemented through the generation of peptide-based vaccines, whereby immunogenic class II peptide fragments from tumor associated antigens such as MUC1 (209), NY-ESO1 (210), MAGE-A3 (211), and HER2 (212) have been injected to induce antigen-specific CD4^+^ T_H_1 response (1). Additionally, research into a universal cancer vaccine examined the efficacy of stimulating CD4^+^ T_H_1 responses through vaccination with promiscuous epitopes from surviving and telomerase proteins (213, 214). Recently, research focus has shifted to generate cancer vaccines through stimulation of CD4^+^ T_H_1 responses to mutated antigens, or neoantigens, selectively expressed by malignant tissue (168), and with the known synergistic effects of tumor specific CD4^+^ and CD8^+^ T cells, peptide epitopes capable of binding both MHC-I and MHC-II show potential to optimize vaccination efficiency (1, 207, 215). In 2017 Ott et al. demonstrated the efficacy of personalized neoantigen vaccines for the treatment of melanoma patients where specific mutations were identified in patients and synthetic long peptides were used in vaccination to stimulate both CD4^+^ and CD8^+^ responses. While CD4^+^ T_H_1 cells demonstrated the highest rates of tumor specific response and targeted 60% of the unique neoantigens used across patients, resulting in no recurrence in four out of six patients 25 months after vaccination, two recurrent disease subsequently treated with anti-PD1 therapy led to complete tumor regression and expansion of the repertoire of neoantigen-specific T cells (1, 86). Similarly, Tondini et al. developed a poly-neoantigen vaccine composed of a fusion gene incorporating three neoepitopes derived from mouse colorectal tumor in combination PD-1 IBC to target both CD4^+^ and CD8^+^ responses in murine colorectal cancer models (216).

The pivotal role of DC to generate T cell mediated tumor immunity *via* activation, priming, and induced rapid expansion of antigen-specific T cells implicates therapeutic potential of DC-targeted immunotherapy development (215). Loading of patient autologous DC with previously identified immunogenic epitopes from tumor antigens and reinfusion of antigen-loaded DC can lead to the induction of specific anti-tumor CD4^+^ T_H_1 responses (1). The first FDA approved DC vaccine (Sipuleucel-T) was approved in 2010 for metastatic prostate cancer (217). Following this, several trials have evaluated the therapeutic benefits of DC vaccines for the treatment of various cancer types (**Table 2**). Findings from multiple trials testing therapeutic efficacy of a DC vaccine primed with WT-1, a TAA overexpressed in glioblastoma, or autologous tumor lysate to treat patients with glioblastoma, has been reviewed by Eagles et al. (218). In 2016, De La Cruz et al. demonstrated the efficacy of a HER2-DC vaccine in HER2^+^ breast cancer patients, where treatment induced a significant increase in anti-HER2 CD4^+^ T_H_1 response and improved rates of pathological complete response (160, 163, 219). A clinical trial (NCT00910650) adoptively transferring MART-1 T cell receptor (TCR) transgenic lymphocytes together with MART-1 peptide-pulsed DC vaccination in HLA-A2.1 patients with metastatic melanoma showed evidence of tumor regression (220).

There are several cancer vaccines showing great promise for the treatment of different cancer types but there are still challenges in using this approach to treat advanced disease, and vaccination alone may be insufficient to control tumor progression. The efficacy of these vaccines is highly dependent on the identification of proper stimulatory antigens and functional status of the individual immune response in patients. Future research can optimize this immunotherapeutic strategy for eliciting tumor-specific CD4^+^ T cell responses by considering antigen dosing and immunogenicity, timing of the therapy, the role of adjuvants, immunosuppressive TME, and combinational strategies (221).

### Adoptive Cell Therapy

Adoptive cell therapy (ACT) involves the generation of tumor specific T cells *ex vivo* that can be reinfused into patients. Clinical success of ACT depends greatly on the expansion of tumor specific T cells ex vivo, homing to the tumor site, and persistence following infusion. Although most cell therapies focus on CD8^+^ CTLs due to their tumor killing capabilities, considering the molecular ‘help’ by CD4^+^ T_H_ cells is required for CD8^+^ cytotoxicity and recruitment, adoptive transfer of CD4^+^ T cells may play an important role in overall tumor immune response (84). Interestingly, transfer of CD8^+^ T cells alone has shown to have low tumor free survival rates whereas transfer of both CD4^+^ T_H_1 cells and CD8^+^ CTLs has shown a synergistic anti-tumor response resulting in complete regression in 80% of mice (222). Previous expansion methods focus primarily on the use of the cytokine IL-2 to expand T cells *ex vivo*. CD4^+^ T cells can be transformed into regulatory T cells in the presence of IL-2 and TGF-β secreted in the TME, allowing immune evasion. A study by K.L. Knutson et al. showed that IL-2 alone resulted in the loss of proliferation of antigen specific CD4^+^ T cells; however, addition of IL-12 was able to overcome the loss of proliferation (223). This suggests there is a pressing need to explore other cytokines that can successfully expand tumor specific CD4^+^ T cells without generating regulatory T cells. Elimination of immune suppressive cells such as MDSC has been shown to greatly enhance the efficacy of ACT (224), suggesting that ACT must also overcome the immunosuppressive effects of the TME and other innate immune cells to amplify the therapeutic efficacy.

Success of ACT also relies heavily on the generation of T cells that can persist long after infusion. K.A. Read et al. showed that IL-7 and IL-15 maintain important memory phenotypes in CD4^+^ T_H_ cells which aid in long term survival (225). IL-7 is also believed to have a role in T cell trafficking to secondary lymphoid organs. Peter Cohen and his group described tumor specific CD4^+^ and CD8^+^ T cells expansion from unfractionated PBMCs using an activator of innate immunity. Addition of toll-like receptor agonists, LPS and R848 (resiquimod), followed by addition of synthetic long peptides (>20aa) derived from widely expressed oncoproteins (MUC1, HER2/neu and CMVpp65) enhanced the processing and presentation of exogenous TAA. Addition of IL-7 enhanced the antigen-driven outgrowth of CD4^+^ and CD8^+^ T cells (226). Therefore, IL-7 and IL-15 can be potential alternatives to using IL-2 in the generation of tumor specific T cells.

Adoptive cell therapy alone is not enough in providing long term effects that can prevent relapse in patients. Combination therapies using immune checkpoint blockade, migratory molecules such as CXCR2, and stimulatory cytokines such as IFN-γ and ACT have been studied widely in melanoma models with great promise (186). Toxicity, however, can be a potential hinderance to combination therapies. Thus, finding a safe adoptive cell therapy that can harness the full effects of both CD4^+^ and CD8^+^ T cells is paramount in future immunotherapies.

### Chimeric Antigen Receptor T Cell Therapy

Chimeric antigen receptor T cell (CAR-T) therapy entails genetic engineering of a patient’s own T cells to express membrane spanning fusion receptors with defined specificities for tumor associated antigens. In humans, CD4^+^ T cells as part of the CAR has been shown to induce target cell apoptosis in an MHC and Fas-independent manner, *via* cytolytic degranulation by perforin and granzyme (227, 228). However, reportedly low granzyme and perforin expression on CD4^+^ T cells, compared to CD8^+^ T cells, may contribute to their limited cytotoxicity (229). Equal tumor cell killing capacity of CD4^+^ and CD8^+^ CAR-T cells, albeit longer conjunction and delayed kinetics in CD4^+^ cells (230), and apoptosis and anergy in CD8^+^ T cells without the molecular help from CD4^+^ T cells in the vicinity suggest CD4^+^ CAR-T can potentiate the effects of the therapy in cancer (231). Between GBM-associated antigen-targeting CD4^+^ and CD8^+^ CAR-T cells, CD4^+^ CAR T cells showed effector persistency after tumor challenge and similarly in orthotopic GBM model, CD4^+^ CAR-T outcompeted CD8^+^ CAR-T in terms of durable anti-tumor response (232). In GBM *in vitro* and *in vivo* models, Brown et al. (233) tested anti-tumor effects of IL13Rα2-specific CAR T cells engineered from purified CD4^+^ or CD8^+^ T_CM_ pools and showed superior tumor killing by CD4^+^ CAR-T cells, along with higher cytokine production and persistent effector function upon tumor challenge, when compared with CD8^+^ CAR-T cells. Intracranial injection of CD4^+^ CAR-T in an NSG model of GBM showed durable anti-tumor efficacy and prolonged survival, while mice receiving CD8^+^ CAR-T cells recurred following an initial response (233). In a preclinical NSG mouse model, administration of CD19-CAR-T using CD4-targeted lentiviral vector (CD4-LV) displayed T_H_1/T_H_2 phenotype of the CAR-T, with a superior and faster tumor killing ability than CD8-LV CAR-T cells alone or in combination with CD4-LV. Such prolonged response by CD4^+^ CAR-T cells in preclinical and clinical models can be attributed to higher exhaustion in CD8^+^ cells (234). Overall, navigating the immune suppressive effects of the TME and reducing clinical toxicities, while maintaining a durable anti-tumor response, will be of paramount importance to successful CAR-T cell therapy for solid tumors.

## Future Prospects of T_H_ Cells in Cancer Immunotherapy

Recent research has underscored the significance of CD4^+^ T_H_ cells as a component of anti-tumor immune response. In this review we discuss why CD4^+^ T_H_ cells are considered an integral component of current immunotherapy research and how the shifting balance between T_H_1 and T_H_2 cells, along with other T_H_ cell subtypes, modulate the intratumoral immune response. Notably, current research has pointed out how these other CD4^+^ T_H_ subtypes, such as immunostimulatory T_H_9, T_fh_ and T_H_17 while immunosuppressive T_H_2, T_H_17, T_reg_ cells and inhibitory function of MDSC, can sway the anti- vs pro-tumorigenic balance of T_H_ immune response and suggest the clinical relevance of targeting these CD4^+^ T_H_ subtypes. Therapeutic intervention to regulate not only T_H_1 and T_H_2 functional response, but other stimulatory/suppressive immune populations may be critical for more efficacious therapy design.

Review of recent literature points out the potential advantages of CD4^+^ T_H_ cell-based immunotherapy in comparison with strategies focused on CD8^+^ T cells. These cells are necessary and sufficient to activate CD8^+^ T cells for amplified anti-tumor response, along with their own contribution to cytotoxicity of tumor cells. The requirement for specific peptide recognition and HLA class matching can limit the therapeutic success of CD8^+^ T cells targeting neoantigens which are primarily derived by point mutations, since those mutations can significantly alter the interaction kinetics with CD8^+^ T cells and diminish CTL activity. The more promiscuous nature of CD4^+^ T cells permits interaction with a broader variety of neoantigens and mutated oncodrivers. Simultaneously, a reciprocal regulation of CD4^+^ T_H_ cells and B cells have been shown to be essential for both T_H_1 and T_H_2 and effector B cell differentiation and function, which hints at the therapeutic potential of stimulating CD4^+^ T_H_ immune response and indirectly boosting antibody secretion by B cells for enhanced anti-tumor response. However, studies in various pre-clinical models and clinical trials that have pointed out potential obstacles such as a hostile TME, presence of inhibitory T cell populations and immune checkpoint receptors suggest that stimulating a single subpopulation of CD4^+^ T_H_ function alone may not be adequate for robust anti-tumor response. Combinations of therapies that can drive multiple subtypes of CD4 T_H_ may better overcome this inadequacy and improve therapeutic efficacy in cancer. Targeted inhibition of oncodrivers by blocking/neutralizing antibodies and small molecule inhibitors, cell cycle kinases CDK4/6 inhibitors and standard-of-care therapeutics in combination with immunotherapy that drive CD4^+^ T_H_ cells are currently being tested in various stages of clinical trials and warrant future research to delve into the mechanism that these therapies have on anti-vs pro-tumorigenic CD4^+^ T_H_ responses for refined synergy. Developing DC-based vaccine platforms to stimulate oncodriver-specific T_H_ immune response can facilitate immunotherapy and future synergistic combination for effective cancer treatment. Understanding the role of immunosuppressive T_H_ cells, such as T_H_2, T_H_17 and T_reg_, in the TME is equally critical to identify nodes in this regulatory network for therapeutic intervention. A successful cancer immunotherapy will require careful balancing of the CD4^+^ T_H_ compartment in order to orchestrate essential efforts that mediate tumor regression. A comprehensive overview of the CD4^+^ T_H_ cells, as discussed in this review, will help to elucidate the framework of CD4^+^ T_H_ function and highlight the clinical relevance of harnessing CD4^+^ T_H_ cells in cancer immunotherapy to encourage future translational research.

## In Memoriam

### Dedicated to Peter Cohen, MD 

Peter Cohen, MD was an early advocate for CD4^+^ T cell therapy for cancer, at a time when most of the field was focused almost exclusively on CD8^+^ CTL. Beginning as a Physician Scientist at UC San Diego, he spent many years at NCI, in both Surgery and Medicine Branches, working on advancing the concept that CD4^+^ T cells were critical to immunotherapy for cancer. Some of his early predictions are being borne out in the recent literature, confirming the critical importance of the CD4^+^ T cell populations in eliminating tumors. Dr. Cohen was also instrumental in developing technologies to facilitate the use of dendritic cells as vaccine platforms for cancer immunotherapy. Dr. Cohen went on to work with Dr. Suyu Shu at the Cleveland Clinic, and for the past 10 years has worked as a medical oncologist at the Mayo Clinic in Arizona. Those of us privileged to have Dr. Cohen as a mentor or colleague were charmed by his irrepressible humor and quick wit, inspired by his intellect and drive, and humbled by his compassion and dedication to the care of his patients. It is in sincere appreciation for Dr. Cohen’s enriching contributions to science, to our lives, and to our careers that we dedicate this article to his memory.

## Author Contributions

ABa, BC, and KK contributed to concept, outline and writing of the review. ABa, GR, GA, CG, ABe, CS, and KK contributed to writing. GK, MD, BC, and KK reviewed and edited the manuscript. All authors contributed to the article and approved the submitted version.

## Funding

This work was supported by Department of Defense (Award# W81XWH-16-1-0385) awarded to BC, GK and MD. This work was also supported by Pennies in action to BC and GK. MD is also supported by the Helen B. Slonaker Endowed Professor for Cancer Research.

## Conflict of Interest

BC and GK have patent application filed for intellectual property on a human version of DC1.

The remaining authors declare that the research was conducted in the absence of any commercial or financial relationships that could be construed as a potential conflict of interest.
